# Unveiling the Chemical Composition, Antioxidant, and Antimicrobial Potentials of *Foeniculum vulgare* Mill: A Combined In Vitro and In Silico Approach

**DOI:** 10.3390/ijms26104499

**Published:** 2025-05-08

**Authors:** Bouchra El Moumen, Amal Bouzoubaa, Aziz Drioiche, Mohamed Eddahmouny, Omkulthom Al Kamaly, Abdelaaty Abdelaziz Shahat, Hanane Touijer, Nadia Hadi, Samira Kharchouf, Ali Cherrat, Kamal Fadili, Hajar El Ouadni, Amina Bari, Touriya Zair

**Affiliations:** 1 Research Team of Chemistry of Bioactive Molecules and the Environment, Laboratory of Innovative Materials and Biotechnology of Natural Resources, Faculty of Sciences, Moulay Ismaïl University, B.P. 11201 Zitoune, Meknes 50070, Morocco; elmoumen.bouchra4@gmail.com (B.E.M.); bouzoubaa.amal@yahoo.fr (A.B.); mo.eddahmouny@edu.umi.ac.ma (M.E.); hananetou@gmail.com (H.T.); nadia.hadi1@gmail.com (N.H.); samirakharchouf@yahoo.fr (S.K.); alicherrat2016@gmail.com (A.C.); k.fadili@edu.umi.ac.ma (K.F.); 2 Higher Institute of Nursing Professions and Health Techniques of Fez, Regional Health Directorate Fez-Meknes, EL Ghassani Hospital, Fez 30000, Morocco; 3 Department of Pharmaceutical Sciences, College of Pharmacy, Princess Nourah bint Abdulrahman University, P.O. Box 84428, Riyadh 11671, Saudi Arabia; omalkmali@pnu.edu.sa; 4 Pharmacognosy Department, College of Pharmacy, King Saud University, P.O. Box 2457, Riyadh 11451, Saudi Arabia; ashahat@ksu.edu.sa; 5 Pharmacodynamics Research Team ERP, Laboratory of Pharmacology and Toxicology, Faculty of Medicine and Pharmacy, University Mohammed V in Rabat, Rabat B.P. 6203, Morocco; elouadni.hajar@gmail.com; 6 Laboratory of Biotechnology, Environment Agrifood and Health, Faculty of Sciences Dhar El Mahraz, Sidi Mohamed Ben Abdellah University, BP 1796 Atlas, Fez 30000, Morocco; aminabari3@gmail.com

**Keywords:** *Foeniculum vulgare* Mill, fenchone, anethole, butyl ferulate, chlorogenic acid, quercetin-3-glucuronide, antioxidant activity, antimicrobial activity

## Abstract

This study on *Foeniculum vulgare* Mill., derived from seeds collected in Meknes (Morocco), evaluates in vitro and in silico the therapeutic potential of its extracts and essential oil through a comprehensive analysis of its phytochemical composition, as well as its antioxidant and antimicrobial activities. Aqueous extracts (E0), hydroethanolic extract (E1) obtained via Soxhlet, decoction (E2), and essential oil (EO) obtained through hydrodistillation were analyzed using HPLC/UV-ESI-MS and GC-MS, revealing a richness in phenolic and terpenic compounds. The quantification of total polyphenols, flavonoids, and tannins in aqueous and organic extracts was performed using spectrophotometric methods. Antioxidant activity was assessed through three methods: DPPH, FRAP, and Total Antioxidant Capacity (TAC). The antimicrobial activity of the essential oil and decoction was evaluated by microdilution in microplate assays. The aqueux extract was dominated by butyl ferulate (14.33%), while hydroethanolic extract contained chlorogenic acid (14.79%) and quercetin-3-glucuronide (13%). The extract (E_2_) was characterized by dihydrocaffeic acid (11.25%) and 3-O-caffeoylshikimic acid (11.08%), whereas the EO was primarily composed of fenchone (24.72%), trans-anethole (22.22%), and limonene (20.48%). Antioxidant assays (DPPH/FRAP/TAC) demonstrated decreasing efficacy as follows: EO exhibited the highest efficiency (IC_50_ = 51.45 μg/mL), followed by E_1_ (93.71 μg/mL), E_0_ (212.86 μg/mL), and E_2_ (397.41 μg/mL), confirming a correlation between phenolic composition and antioxidant activity. Furthermore, antimicrobial tests highlighted a pronounced fungicidal effect against *Candida albicans* (MIC = 3.13 mg/mL) and *Aspergillus niger* (6.25 mg/mL), contrasting with a more moderate inhibition of *Escherichia coli* and *Staphylococcus aureus*. Molecular docking simulations identified stable interactions between chlorogenic acid, quercetin-3-glucuronide, and microbial proteases, suggesting a synergistic inhibitory mechanism. This research validates the potential of *F. vulgare* as a source of bioactive molecules with promising applications in phytotherapy for managing oxidative stress and fungal infections, while emphasizing the need for clinical studies to confirm these effects in vivo.

## 1. Introduction

*Foeniculum vulgare* Mill., commonly known as fennel, is a herbaceous plant belonging to the Apiaceae family, widely recognized for its numerous medicinal, culinary, and industrial applications. The genera within this family are characterized by strong flavors and aromas due to the presence of schizogenous ducts containing oil, mucilage, and resins, which can be found in both the aerial parts (leaves, stems, and fruits) and the roots [1]. This distinctive feature endows the plants of the Apiaceae family, including *Foeniculum vulgare*, with a wealth of secondary metabolites, such as coumarins, flavonoids, saponins, and terpenoids, making them highly suitable for various sectors as follows: food (nutrition, beverages, and spices), pharmaceutical, and cosmetic industries [2]. Furthermore, many species within this family are used in traditional medicine for the treatment of gastrointestinal, reproductive, and respiratory disorders [3,4].

*Foeniculum vulgare* is a perennial plant with delicate, feathery, and almost filamentous foliage, capable of reaching up to 2 m in height. It bears a morphological resemblance to dill. Its leaves, striated and three to four times pinnate, feature filiform segments measuring up to 4 cm in length. The small yellow flowers are grouped into broad, flat umbels, with the flowering period extending from July to October. The fruits, oblong to ovoid in shape, measure 3 to 5 mm in length, and the seeds mature between September and October [1,5]. These dry fruits are widely used in culinary applications [6]. This species grows naturally in coastal regions of the Mediterranean but has become extensively naturalized in various parts of the world, thanks to its adaptability to diverse climates and its resilience to dry, sunlit soils [7]. Its status as a cosmopolitan plant is also attributed to its historical use in traditional medicine and gastronomy, notably as a seasoning.

The use of *F. vulgare* dates back to antiquity, where it was cultivated by Egyptians, Chinese, Indians, and Romans, particularly for its aromatic seeds and edible shoots [8]. The Romans utilized fennel, not only for its seeds but also for its succulent stems, which remain a commonly consumed vegetable in southern Italy [9]. All parts of the plant, including its roots, stems, leaves, and fruits, are aromatic and can be employed in various ways. Fennel fruits, as well as its essential oils, serve as flavoring agents in food products, such as liqueurs, bread, pickles, pastries, and cheeses [10,11,12], while also being used in the formulation of cosmetic and pharmaceutical products [13,14].

Studies have demonstrated that *F. vulgare* is effective in addressing a wide range of infectious disorders of bacterial, fungal [15,16], viral, mycobacterial, and protozoal origins [17]. Fennel essential oils have been reported to possess anti-inflammatory, antispasmodic, antiseptic, antithrombotic, antitumor, chemopreventive, cytoprotective, hepatoprotective, hypoglycemic, estrogenic, carminative, diuretic, and analgesic properties [18,19,20], along with beneficial effects in the treatment of gastrointestinal [21] and neurological disorders [22]. The essential oil of *F. vulgare* exhibits significant antimicrobial activity against both Gram-positive and Gram-negative bacteria [23], as well as against yeasts, although its efficacy varies depending on the phase (liquid or vapor) and the concentration used [24]. Moreover, this essential oil possesses strong antioxidant properties, including the ability to scavenge free radicals and enhance the activity of antioxidant enzymes, such as superoxide dismutase, catalase, and glutathione peroxidase [25,26]. Additionally, the essential oil of *F. vulgare* has shown hypoglycemic effects in diabetic rats, significantly reducing blood glucose levels and improving the activity of antioxidant enzymes [27]. Furthermore, certain publications have highlighted that *F. vulgare* possesses a particular effect on memory enhancement and can reduce stress [28].

Although the phytochemistry and antimicrobial properties of fennel, particularly its essential oils, have been studied in a fragmented manner, the available data remain insufficient to fully exploit the potential of this plant. In this context, our study stands out for its in-depth and innovative approach, aiming to uncover the richness of Moroccan *F. vulgare*. By combining a detailed analysis of its phytochemical compounds with a rigorous evaluation of its antioxidant and antimicrobial capacities, both for its essential oils and three distinct types of extracts, we make a significant contribution to the understanding and valorization of this species in key fields, such as health, cosmetics, and the food industry.

## 2. Results

### 2.1. Quality Control of Plant Material

Table 1 summarizes the results of the plant material quality control. *F. vulgare* seeds have a moisture content of around 25.12%, an acid pH of 5.5, and contain 6.4% mineral matter and 93.6% organic matter. The dosage of metallic trace elements showed fairly low contents of arsenic (0.0058 mg/g), chromium (0.0008 mg/g), antimony (0.0023 mg/g), copper (0.003 mg/g) while lead, cadmium and titanium are undetectable against a slightly high iron content (0.271 mg/g) (Table 2). It should be noted that these results are below the limit values for each metallic trace element.

### 2.2. Phytochemical Screening of Plant Material

The phytochemical screening highlighted the richness of this plant in vitamins, minerals, primary and secondary metabolites. Table 3 displays the composition of primary and secondary metabolites in *F. vulgare*. It was observed that *F. vulgare* is abundant in lipids (sterols and triterpenes), proteins, reducing sugars, and carbohydrates (oses and holosides). Concerning secondary metabolites, the species is rich in polyphenols, such as flavonoids (leucoanthocyanins, flavones) and tannins alkaloids, mucilages, as well as saponosides. These results are in line with those reported by Kooti et al. (2015) [29] and demonstrate the plant’s richness in components known for their medicinal, culinary, and dietary benefits.

### 2.3. Contents of Polyphenols, Flavonoids and Condensed Tannins

**Figure 1:**
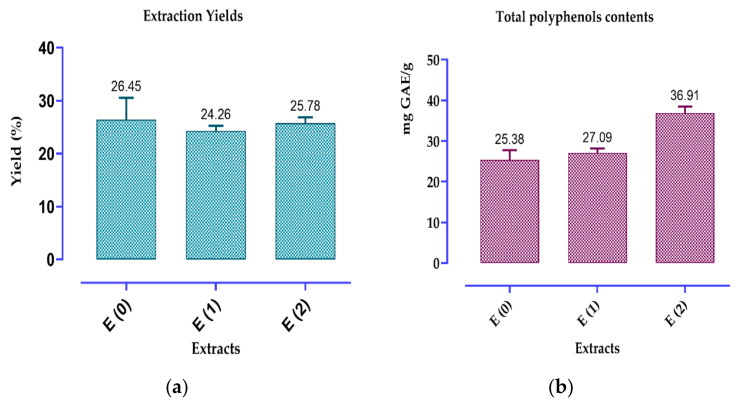

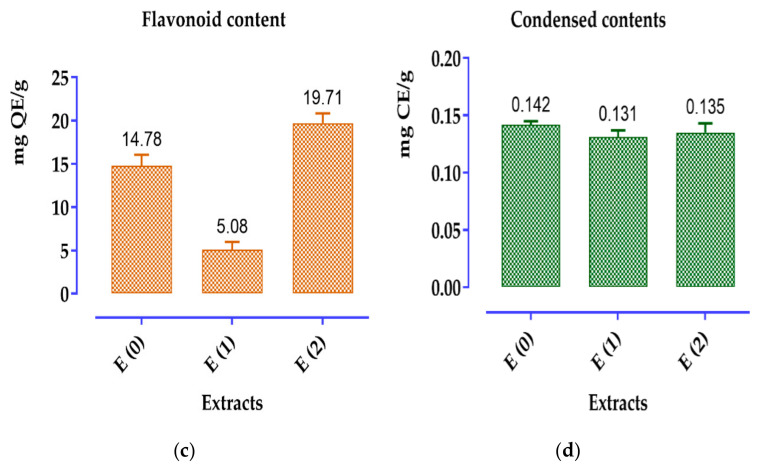
Extraction yield (**a**) and contents of polyphenols (**b**), flavonoids (**c**) and catechic tannins (**d**) in *F. vulgare* extracts. Values denote the mean ± standard deviation based on three separate experiments.

The yields of the various fennel seed extracts using the three methods E (0), E (1) and E (2), are shown in Figure 1a. It can be seen that the extracts obtained by the methods used gave fairly high and almost similar yields, of the order of 26.45%, 25.78%, and 24.26%, respectively. Our results concerning the yield are much higher than those obtained by Anwar et al. (2009) [30], which brought in maximum yields of 15.63% and 6.21%, respectively, from fennel seeds using the ethanol and methanol. Figure 1b–d also present the composition of *F. vulgare* in total polyphenols, flavonoids and condensed tannins. Figure 1b illustrates the total polyphenol content, which are 36.91, 27.09 and 25.38 mg EQ AG/g ES, respectively, for E (2), E (1) and E (0). While for flavonoids, the highest contents were 19.71; 14.78 mg EQ AG/g ES and the lowest were of the order of 5.08 mg EQ AG/g ES recorded, respectively, by E (2), E (0) and E (1) (Figure 1c). Whereas for condensed tannins, the contents are much lower, and were around 0.142; 0.135; 0.131 mg EQC/g ES, respectively, in E (0), E (2) and E (1) (Figure 1d). These differences in the quantity of total polyphenols, flavonoids and catechin tannins, may be due to the varying effectiveness of solvent extraction [31].

### 2.4. Identification of the Chemical Composition of Phenolic Compounds from the Extracts of F. vulgare by LC/UV

**Figure 2:**
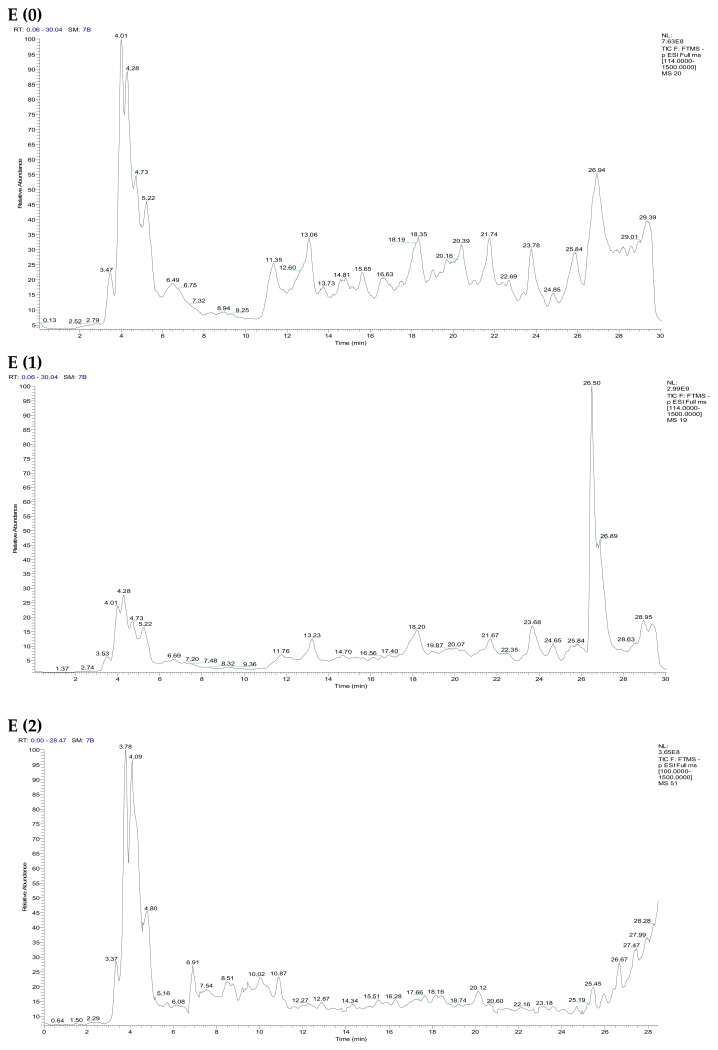
HPLC/UV-ESI-MS chromatogram of *F. vulgare* extracts.

The chemical analysis of *F. vulgare* extracts was carried out using the HPLC/UV-ESI-MS technique, enabling a detailed characterization of the compounds present in the chromatograms shown in Figure 2. In-depth interpretation of the mass spectra in negative ionization mode, coupled with examination of the chromatograms, led to the identification of 65 molecules, listed in Supplementary Table S1. The three extraction methods used—aqueous Soxhlet extraction (E (0)), hydroethanolic Soxhlet extraction (E (1)), and decoction (E (2))—revealed distinct phytochemical profiles. The aqueous extract E (0) showed the presence of 28 bioactive compounds, dominated by phenolic acids (Table 4), which represent 49.96% of the total content. The major compound identified in E (0) is butyl ferulate, which accounts for 14.33% of the extract. The hydroethanolic extract E (1), obtained with an ethanol/water mixture (70:30), was distinguished by its greater molecular diversity, with 34 compounds identified. Phenolic acids also dominate this extract, representing 33.39% of the total content, while flavonoids make up a notable proportion of 42.91% (Table 4). Among the major compounds, chlorogenic acid (14.79%) and quercetin-3-glucuronide (13%) are particularly abundant. Finally, the decocted extract E (2) allowed the identification of 37 bioactive compounds, with a predominance of phenolic acids (37.3%). However, this extract stands out due to its richness in lignans (7.82%), phenolic diterpenes (8.08%), and dipeptides (5.42%), suggesting that boiling promotes the extraction of thermostable or weakly polar compounds. The major compounds in this extract include dihydrocaffeic acid (11.25%) and 3-O-caffeoylshikimic acid (11.08%). These results highlight the influence of extraction conditions on the phytochemical profile of the extracts. They particularly emphasize the effectiveness of the hydroalcoholic solvent with intermediate polarity in extracting a diverse range of secondary metabolites, while also illustrating the complementarity of the different approaches.

**Figure 3:**
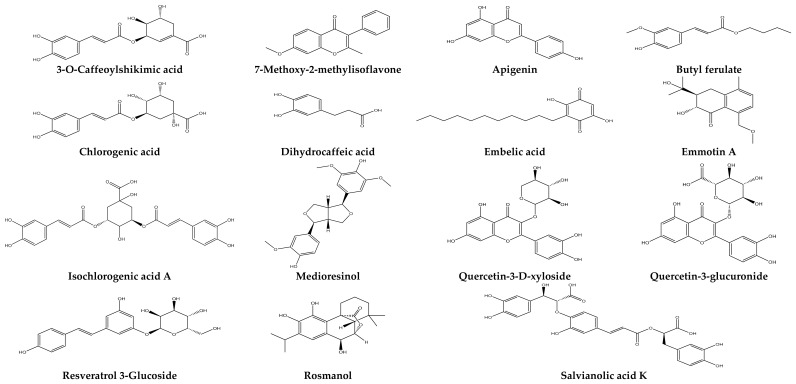
Structures of the compounds identified in the extracts of *F. vulgare*.

The combined HPLC/UV-ESI-MS analysis of *F. vulgare* extracts allowed for the fine and structured characterization of the fragmentation profiles of bioactive compounds, providing a deep understanding of their molecular architecture. The observed fragmentation mechanisms are specific to each chemical class and reveal essential diagnostic signatures for structural validation. Lignans, exemplified by medioresinol ([M−H]^−^ = 387), fragment into ions at *m*/*z* 207 and 179, indicating a classical breakage of ether bonds between phenylpropanoid units. Phenolic acids, such as embelic acid, dihydrocaffeic acid ([M−H]^−^ = 181), and 3-O-caffeoylshikimic acid ([M−H]^−^ = 335), show typical losses of CO_2_ (*m*/*z* 137) or caffeic residues (*m*/*z* 291, 197), characteristic of carboxylic chain or ester bond cleavages. Chlorogenic acid ([M−H]^−^ = 353) and iso-chlorogenic acid A ([M−H]^−^ = 515) generate fragments at *m*/*z* 191 and 179 (quinic acid and caffeic acid, respectively), confirming their nature as acylquinates. Phenolic diterpenes like rosmanol ([M−H]^−^ = 345) produce an ion at *m*/*z* 283, suggesting isoprene loss or dehydration, while sesquiterpenoids like emmotine A ([M−H]^−^ = 277) reveal fragments at *m*/*z* 243 due to terpene skeleton degradation. Glycosylated polyphenols, such as resveratrol-3-glucoside ([M−H]^−^ = 389) and quercetin-3-glucuronide ([M−H]^−^ = 477), undergo losses of 162 u (glucose) and 176 u (glucuronide), revealing fragment ions at *m*/*z* 227, 301, and 151, typical of flavonoid fragmentation. Aglycone flavonoids, such as apigenin ([M−H]^−^ = 269) and 7-methoxy-2-methylisoflavone ([M−H]^−^ = 265), show breakage of the C-ring and decarboxylations, leading to ions at *m*/*z* 227, 237, 179, and 159. Esters like butyl ferulate ([M−H]^−^ = 249) lose a butyl group (*m*/*z* 193), while more complex polyphenols like salvianolic acid K ([M−H]^−^ = 555) reveal multi-stage fragmentation at *m*/*z* 493, 313, and 179, indicating a structure rich in caffeic units. These fragmentation patterns, specific and reproducible, serve as powerful tools for structural identification, chemical classification, and confirmation of annotations in complex plant extracts. They also highlight the metabolic richness of *F. vulgare* and reinforce the pharmacognostic interest of this medicinal species. Figure 3 illustrates the structures of the major identified compounds, providing essential information for a thorough structural analysis.

*F. vulgare* extracts are distinguished by their richness in bioactive compounds, particularly polyphenols and phenolic acids, which provide potent antioxidant and antimicrobial properties. These findings support the work of [32], as well as [33,34], which highlighted the key role of these compounds in the biological activities of this plant. The ethanolic extract, known for its effectiveness in extracting flavonoids and polyphenols, stands out for its ability to concentrate bioactive compounds, such as quercetin-3-glucuronide, quercetin-3-D-xyloside, apigenin, and rutin. These molecules, widely documented for their protective properties at the cellular level, have been highlighted in various studies [35]. Additionally, the notable presence of chlorogenic acid and iso-chlorogenic acid A provides promising potential for antimicrobial applications, as confirmed by the studies of Khlood et al. (2023) [36] and Mingsan et al. (2020) [37]. Although the decoction extract is less concentrated in flavonoids, it remains effective for extracting certain polyphenols and phenolic acids while offering better bioavailability for certain molecules. Thus, these results emphasize the relevance of *F. vulgare* extracts for various applications in the pharmaceutical and food industries, due to their remarkable functional properties.

### 2.5. Yields and Quality Control of EO

The yield of fennel seed EO is shown in Table 5. The yield of hydro-distilled fennel seed essential oil is 2.5%. The results obtained in the present study are in line with those reported by other researchers, who found yields between 2.82 and 3.38% using hydro-distillation [31,38]. This yield is higher than that obtained by Diao et al., 2014 [23], which recorded a yield of 1.74%, while the yield of EO and ethanol extract of fennel seeds from Portugal were 0.1% and 6.9%, respectively [30].

### 2.6. Chemical Composition of F. vulgare Seed EO

**Figure 4:**
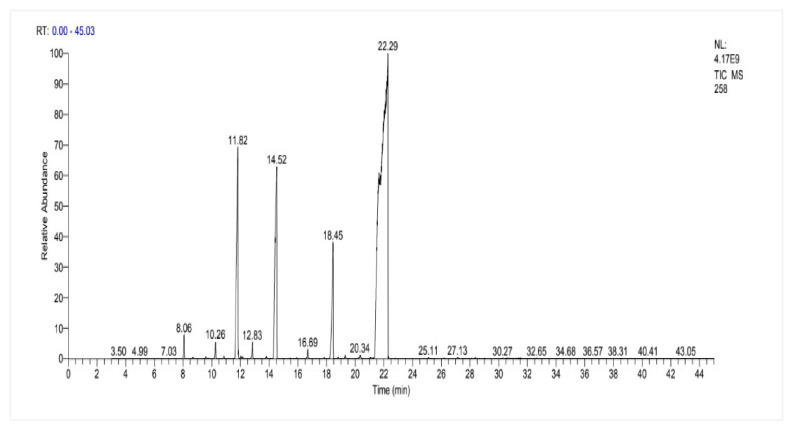
Chromatogram (GC-MS) of the EO of *F. vulgare* seeds.

**Figure 5:**
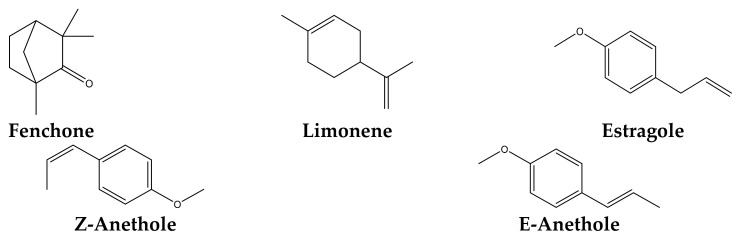
Structures of the major compounds identified in the EO of *F. vulgare*.

The results of the chemical composition of fennel essential oil are shown in Table 6. A total of 25 chemical compounds representing approximately 99.99% of the total EO composition of *F. vulgare* leaves were identified using gas chromatography-mass spectrometry (GC-MS) (Figure 4). The main constituents of the EO tested were fenchone (24.72%), followed by trans-anethole (22.22%), limonene (20.48%), cis-anethole (19.18%) and Methylchavicol (estragole) (8.79%) (Figure 5, Table 6). The illustrated results also showed the presence of two chemical families at high levels, specifically oxygenated monoterpenes (77.02%) and monoterpenes (22.90%). In addition, the fennel EO tested also contained considerable quantities of various minor constituents whose contribution was less than 1%, such as sesquiterpenes (0.07%). Regarding the chemical constituent groups represented, cis/trans-anethole, fenchone, and estragole were the major oxygenated monoterpenes, while limonene was the major monoterpene. In this regard, a comparative profile has been reported by several researchers who revealed the presence of trans-anethole, fenchone, estragole and limonene as the main components of the EOs of *F. vulgare* fennel seeds with percentages depending on the region (Podgorica, central Montenegro, Turkey) [15,39,40]. The variation in the chemical composition of EO from one country to another could be due to agro-climatic conditions (climate, season, geographic) among many more factors that influence the adaptive metabolism of plants [41].

Due to its hypolipidemic and antiatherogenic activities, this plant could be used to control cardiovascular disorders as stated by Garga et al. (2009) [42]. Fennel’s high polyphenol content means that it may play an essential role in cancer chemoprevention. The anethole contained in its seeds has an inhibitory effect on the activation of TNF-α by the transcription factor NF-KB. Studies have shown that anethole also inhibits cellular responses induced by such cytokines, which could explain its role in cancer protection [31]. As with green anise, Chinese star anise and tarragon, the main active ingredient in both fennel varieties is anethole, which makes up around 80% of the EO [43]. Bitter fennel is also rich in fenchone, while sweet fennel contains more estragole [41]. Laboratory experiments have demonstrated anethole’s antibacterial and antimycotic properties (against microscopic fungi) [44,45].

Fenchone is a monoterpene occurring in the EOs of various plants, including *F vulgare* [46,47]. The studies conducted by Pessoa et al. (2020), Araruna et al. (2025), and Aćimović et al. (2025) have substantiated the anti-inflammatory, antioxidant, wound-healing, antidiarrheal, antifungal, antinociceptive, and bronchodilatory properties of fenchone [48,49,50]. It is thought to possess antispasmodic properties, helping to relieve stomach aches and painful menstruation [51]. Additionally, the anti-tumorigenic effects of D-limonene have been widely studied across various cancer types [52].

**Figure 6:**
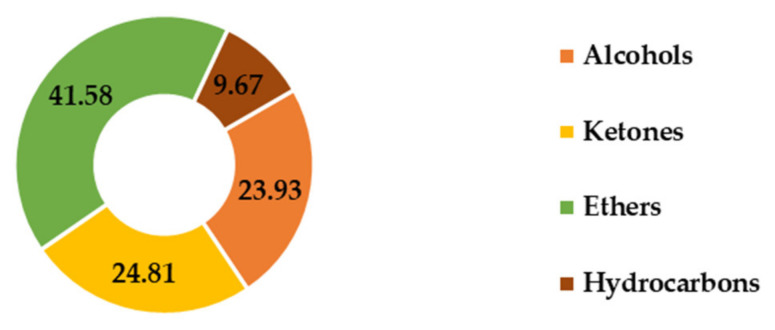
Distribution of families of chemical compounds of *F. vulgare* EO.

A comprehensive analysis of the chemical composition of the essential oil (EO) revealed that *F. vulgare* EO is predominantly composed of oxidized ethers (41.58%), followed by ketones (24.81%) and hydrocarbons (23.93%), with alcohols present in lower amounts (9.67%). Notably, other chemical groups, such as aldehydes and epoxides, are entirely absent from the EO of this plant (Figure 6).

### 2.7. Antioxidant Activities

**Figure 7:**
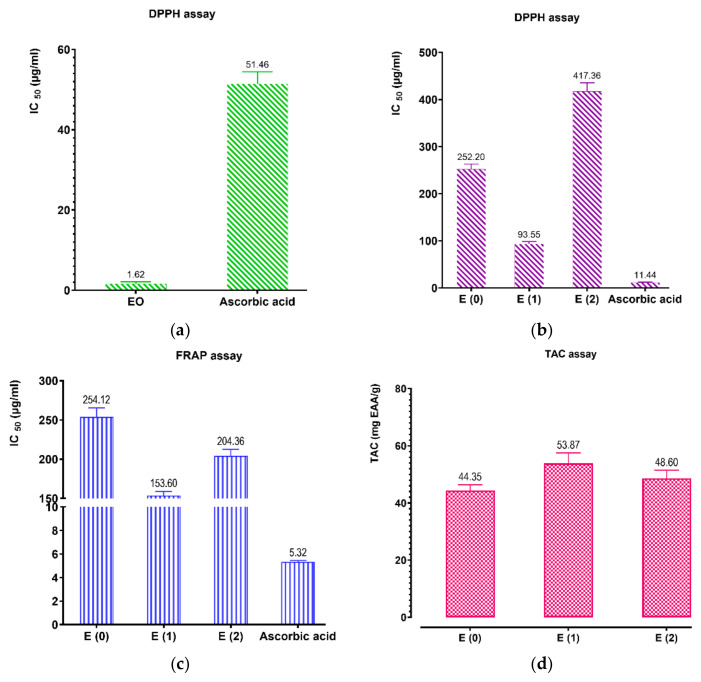
Antioxidant activity of the EO and extracts by DPPH assay (**a**) and (**b**), respectively. Antioxidant activity of the extracts by FRAP assay (**c**) and TAC assay (**d**). Mean values ± standard deviations of determinations performed in triplicate are reported. Means are significantly different (*p* < 0.001).

We examined the free radical neutralizing activity and lipid oxidation inhibition of fennel seed essential oil and the three extracts. Free radicals, which are involved in the lipid peroxidation process, are considered to play a major role in many chronic pathologies, such as cancer and cardiovascular disease among others, hence the need to neutralize them. The free radical neutralizing activities of EO (Figure 7a) and *F. vulgare* extracts (Figure 7b) were measured by the DPPH assay. *F. vulgare* seed extracts showed excellent radical-neutralizing activity, with IC_50_ values (the extract concentration providing 50% of inhibition) of 212.86; 93.71; 397.41 μg/mL for E (0), E (1), E (2) extracts, respectively (Figure 7). Still according to the IC_50_ values grouped together in Figure 7a, the antioxidant power of *F. vulgare* EO is 51.45 μg/mL, which is higher than that of the standard antioxidant ascorbic acid. In comparison with the antioxidant activity of the essential oil and extracts, the antioxidant power of the E (2) extract is lower than that of the other extracts and the EO. Moreover, the E (1) extract has a higher antioxidant activity than the E (0) and E (2) extracts. However, Anwar et al. (2009) [30] and Goswami et al., 2014 [53], reported IC_50_ values between 23.61 and 83 μg/mL, using methanol and ethanol extracts, which are considerably lower than the results achieved with the extracts used. The free radical scavenging activity of fennel extracts could be due to their higher content of phenolic components. These hydroxyl phenolic compounds can donate hydrogen atoms to DPPH and trap it [54].

The reduction activity of ferric iron to ferrous iron was carried out on the E (0), E (1) and E (2) extracts of *F. vulgare*, as well as on ascorbic acid, the standard used as a reference. The results show that the reducing power of iron is proportional to the increase in concentration of the three extracts studied. The concentrations that provide 50% inhibition (IC_50_) were calculated from the curve in Figure 7c. The results showed that the IC_50_ value of ascorbic acid has a higher antioxidant power (5.32 µg/mL). Then the IC_50_ values of the extracts: E (1) (153.6 µg/mL), E (2) (170 µg/mL) and E (0) (254.12 µg/mL), revealed a less significant antioxidant power (Figure 7c). This antioxidant activity of the extracts may have a link with the chemical constituents of these extracts. Indeed, butyl ferulate (14.33%), chlorogenic acid (14.79%) and Dihydrocaffeic acid (11.25%), which are the major compounds in the extracts of *F. vulgare*, revealed a strong correlation with the antioxidant activity.

The ferric-to-ferrous iron reduction assay was performed on three extracts of *F. vulgare*: the aqueous extract obtained via Soxhlet (E_0_), the hydroethanolic extract obtained via Soxhlet (E_1_), and the decoction extract (E_2_), alongside ascorbic acid, which served as the reference standard. The results indicate that the iron-reducing capacity of the extracts increases proportionally with their concentration. The half-maximal inhibitory concentration (IC_50_) values were determined from the dose–response curve shown in Figure 7c. Ascorbic acid demonstrated the strongest antioxidant activity with an IC_50_ value of 5.32 µg/mL. In comparison, the IC_50_ values of the extracts: E (1) (153.6 µg/mL), E (2) (170 µg/mL), and E (0) (254.12 µg/mL), demonstrate a relatively modest antioxidant capacity. The observed antioxidant activity of the extracts is likely attributable to their chemical composition. Notably, key compounds, such as butyl ferulate (14.33%), chlorogenic acid (14.79%), and dihydrocaffeic acid (11.25%) in *F. vulgare* extracts, have been shown to exhibit a strong correlation with antioxidant activity [55].

The results of the antioxidant activity, obtained through the total antioxidant capacity test described in Figure 7d, indicate that the hydroethanolic extract obtained by Soxhlet presents the highest antioxidant activity (53.87 mg EAA/g), followed by the decoction extract (48.60 mg EAA/g), and finally, the aqueous extract obtained by Soxhlet, which shows the lowest activity (44.35 mg EAA/g). Therefore, the hydroethanolic extract (E (1)) stands out as the most effective in terms of antioxidant activity, likely due to specific compounds or conditions associated with this extract.

### 2.8. Antimicrobial Activity of Essential Oils and Extracts of F. vulgare

For several years, many studies have focused on EOs and medicinal plant extracts to inhibit the growth of microbes [31]. In this study, the antimicrobial activity of fennel (*Foeniculum vulgare*) essential oil and extracts was assessed against a panel of ten clinically relevant pathogenic strains of public health importance, namely the following: *Enterobacter cloacae*, *Klebsiella pneumoniae*, *Escherichia coli*, *Staphylococcus aureus* (BLACT), *Staphylococcus epidermidis*, *Candida albicans*, *Candida dubliniensis*, *Candida tropicalis*, *Candida parapsilosis*, and *Aspergillus niger*. The results of the minimum inhibitory concentrations (MICs) for fennel essential oil ranged from 3.13 to 50 mg/mL across the tested strains, underscoring its antimicrobial potential (Table 7). Notably, the lowest MICs were observed against *Candida albicans* (3.13 mg/mL) and *Aspergillus niger* (6.25 mg/mL), whereas higher concentrations, such as 50 mg/mL, were required to inhibit *Staphylococcus aureus* (BLACT) and *Staphylococcus epidermidis*. These findings suggest that antimicrobial efficacy depends on both the targeted microorganism and on the complex chemical composition of the EO. These results are consistent with those previously reported by Roby et al., 2013 [31], who observed comparable antimicrobial effects against *Escherichia coli*, *Candida albicans*, and *Staphylococcus aureus*. Furthermore, Anwar et al., 2009 [30] also highlighted the antifungal activity of fennel essential oil against *Aspergillus* species, thereby supporting the findings presented herein. Regarding the extracts, the hydroethanolic extract obtained by Soxhlet extraction (E1) exhibited broad-spectrum activity against all tested bacterial and fungal strains. The lowest MICs were recorded against *Candida parapsilosis* (0.78 mg/mL) and *Enterobacter cloacae* (12.5 mg/mL), followed by moderate activity against *Aspergillus niger* and *Klebsiella pneumoniae* (25 mg/mL). The E0 extract also demonstrated antimicrobial activity, with MICs of 50 mg/mL for most strains, except for *Klebsiella pneumoniae* and *Escherichia coli*, which were found to be resistant. In contrast, extract E2 showed limited activity, being effective only against *Enterobacter cloacae*, *Staphylococcus aureus* (BLACT), and *Candida albicans*, with an MIC of 50 mg/mL (Table 7).

Food spoilage and foodborne pathogens can compromise the nutritional quality of foods, causing biochemical changes, weight loss, as well as toxicity that harm human health [56]. EOs, which are volatile, odoriferous compounds from plant metabolism, find several applications in food flavoring and preservation [57]. Recent research has emphasized the antimicrobial potential of monoterpene and sesquiterpene hydrocarbons, along with their oxygenated derivatives, which are the primary constituents of EOs [58,59,60]. Such findings support our study, as fennel seed EO, which contains these components, demonstrates capacity as a natural antimicrobial agent. Fennel is traditionally well known to treat various infectious diseases caused by bacteria, fungi, viruses, and mycobacteria [61,62]. Several studies have already supported the antimicrobial effects of EOs [63]. The data here showed that the extracts and fennel EO were effective against the tested microorganisms, confirming fennel’s antimicrobial and antifungal properties. This consequently suggests its extract could be used to combat multiple antibiotic-resistant bacteria. Such findings are in line with previous studies on fennel EOs results [64]. Several researchers have linked these activities to the presence of terpenes, phenols, aldehydes and ketones as the main components of EOs and, based on the GC-MS results. Our results are confirmatory and attribute the antimicrobial activity obtained in this study to the presence of such compounds [65]. In addition to antimicrobial activities, EO and various fennel extracts have shown good antioxidant activities [65]. Fennel is known as an excellent source of natural antioxidants, owing to its high polyphenol and flavonoid content. Phenolic compounds, such as caffeoylquinic acid and rosmarinic acid, have been proven to have very good antioxidant potential [66,67].

### 2.9. Molecular Docking

This computational study thoroughly examined the antimicrobial and antioxidant properties of the primary chemical compounds found in fennel. The results, summarized in Table 8, highlighted significant differences between phenolic extracts and essential oil components. The antimicrobial potential of fennel compounds was tested against the following ten critical microbial proteins: 7TI1, 3RAE, 4DUH, 2W9S, 1JIJ, 3KP5, 7RJB, 5V5Z, 4YBF, and 4ZA5. Among the phenolic extracts, isochlorogenic acid A demonstrated outstanding affinity, especially with 3KP5 (−10.3 kcal/mol), followed by salvianolic acid K and quercetin-3-D-xyloside, which also exhibited strong interactions with 3KP5 (−9.5 kcal/mol) and 3RAE (−9.9 kcal/mol), respectively. These findings underscore the remarkable antimicrobial potential of phenolic compounds, particularly against targets like 3KP5 and 3RAE. In contrast, monoterpenes from the essential oil, such as trans-anethole, limonene, and fenchone, demonstrated weaker affinities, with binding scores ranging between −4.2 and −6.9 kcal/mol. Chlorogenic acid, while showing moderate interactions with 3RAE (−9 kcal/mol) and 3KP5 (−8.5 kcal/mol), was less effective compared to other phenolic compounds. Rosmanol displayed strong selectivity, with a high affinity for 3RAE (−9.9 kcal/mol) but a weaker interaction with 3KP5 (−6.8 kcal/mol). Lastly, quercetin-3-glucuronide exhibited similar activity to quercetin-3-D-xyloside, though slightly less effective, particularly against 3RAE and 7RJB. These results further highlight the prominent role of flavonoids in the antimicrobial activity of fennel.

The antioxidant activities were evaluated against the following five oxidative targets: 5QJ2, 3NRZ, 1OG5, 1N8Q, and 2CDU. Salvianolic acid K showed a particularly strong affinity for 3NRZ (ΔG = −10.9 kcal/mol) and 1OG5 (ΔG = −10.0 kcal/mol), suggesting an exceptional ability to interact with proteins involved in oxidative stress pathways. Apigenin and rosmanol also demonstrated remarkable performance, with scores below −9 kcal/mol for several targets, thereby confirming their crucial role in antioxidant activity. Additionally, mediorsinol showed a marked selectivity for 5QJ2 (ΔG = −9.0 kcal/mol), suggesting a specific mechanism of action. In contrast, the terpenic compounds present in the essential oil, such as fenchone, limonene, and trans-anethole, exhibited significantly lower affinities (ΔG ≥ −6.5 kcal/mol), indicating that they contribute secondarily to the overall antioxidant activity of fennel. These results highlight that phenolic extracts, particularly salvianolic acid K and apigenin, play a key role in neutralizing free radicals and modulating redox pathways, while the terpenes in the essential oil, though effective, exert a more moderate antioxidant effect.

#### 2.9.1. Interaction with Antibacterial Proteins

This research identifies specific molecular interactions between several phenolic compounds and two main bacterial targets as follows: DNA topoisomerase 4 (3RAE) and the transcriptional regulator TcaR (3KP5), as shown in Table 9. Chlorogenic acid and iso-chlorogenic acid A form hydrogen bonds with topoisomerase residues DG H:1 and DT F:7, while Quercetin-3-D-xyloside creates two hydrogen bonds (ARG A:28, ASP C:510) along with a hydrophobic interaction (HIS A:76). Quercetin-3-glucuronide interacts with DT F:7 and SER A:80, and both Rosmanol and Salvianolic acid K bind through hydrophobic interactions (ALA A:115/PRO A:113 and ALA A:29, respectively), impairing bacterial replication. Additionally, these compounds influence TcaR: chlorogenic and iso-chlorogenic acids via hydrophobic interactions (ALA B:24/ALA B:38), quercetins through hydrogen bonds (ASN B:20), and Rosmanol/Salvianolic acid K through hydrophobic interactions (ALA B:38 and ILE A:9/LEU A:12/VAL B:19/MET B:114/ALA B:118), which may alter bacterial gene expression. These findings highlight a dual action mechanism: enzymatic inhibition and transcriptional modulation, demonstrating the potential of these compounds as broad-spectrum antimicrobial agents.

#### 2.9.2. Interaction with Antioxidant Proteins

The study reveals specific molecular interactions between phenolic compounds and two key oxidative stress enzymes, as described in Table 10. Regarding xanthine oxidase (3NRZ), apigenin forms hydrogen bonds with ARG L:606 and ARG J:32, as well as π-Alkyl interactions with LEU J:41 and PRO L:675, while chlorogenic acid and iso-chlorogenic acid A establish hydrophobic interactions with VAL C:591/VAL L:591 and ARG J:37, respectively. Rosmanol combines a hydrogen bond (THR A:24) and a π-Anion interaction (ASP A:21), whereas salvianolic acid K interacts via VAL L:591 and PRO L:597. For cytochrome P450 2C9 (1OG5), apigenin shows hydrogen bonds (SER A:343, LYS B:421), a π-Anion interaction (ASP A:349), and a π-Alkyl bond (LYS B:420). Chlorogenic and iso-chlorogenic acids present Alkyl interactions with LYS B:423 and ALA A:439, rosmanol displays π-T-shaped contacts (PHE A:419) and π-Alkyl interactions (LYS A:421), and salvianolic acid K forms a π-Alkyl bond with LYS B:420. These findings demonstrate a dual mechanism of xanthine oxidase inhibition and cytochrome P450 2C9 modulation, suggesting that these compounds could act as natural antioxidants by reducing free radical production and neutralizing reactive oxygen species, thus offering promising prospects for the prevention of oxidative stress-related diseases.

**Table 9:** Two-dimensional and three-dimensional interactions of *F. vulgare* compounds with target proteins associated with antimicrobial activities.

Molecules\Proteins	3RAE		3KP5	
	2D	3D	2D	**3D**
Chlorogenic acid	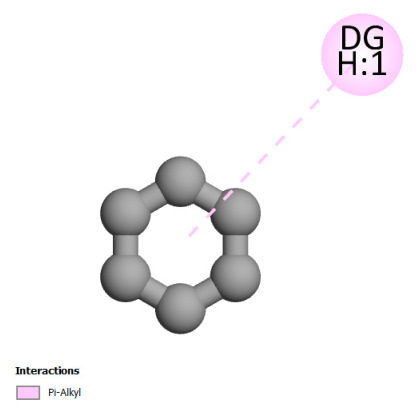	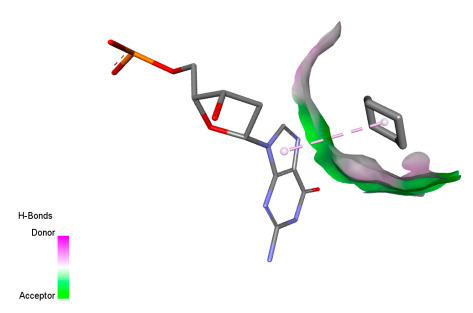	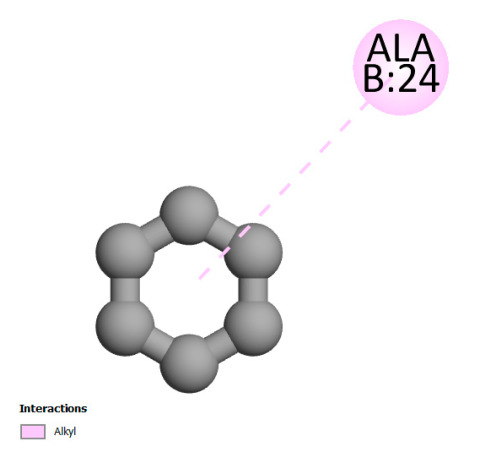	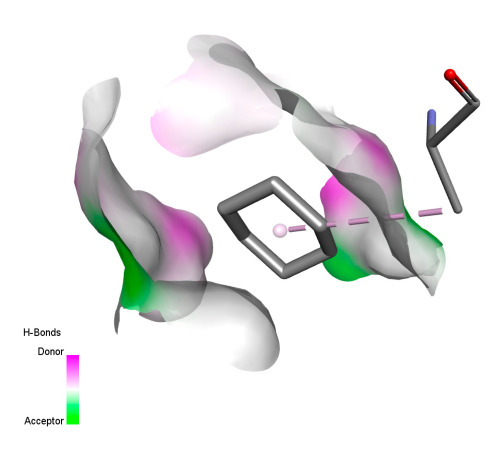
Isochlorogenic acid A	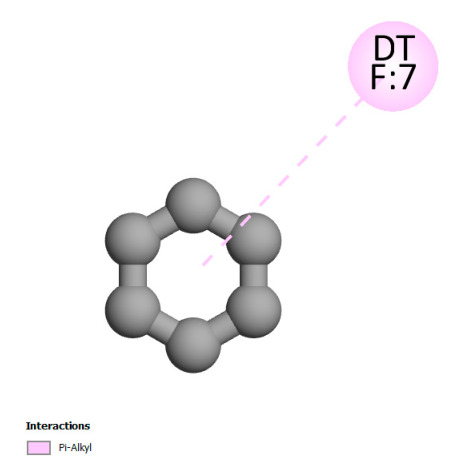	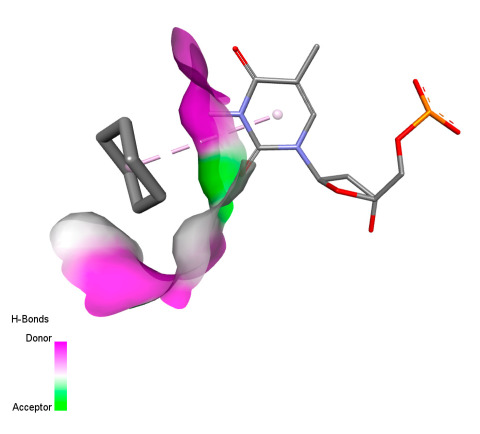	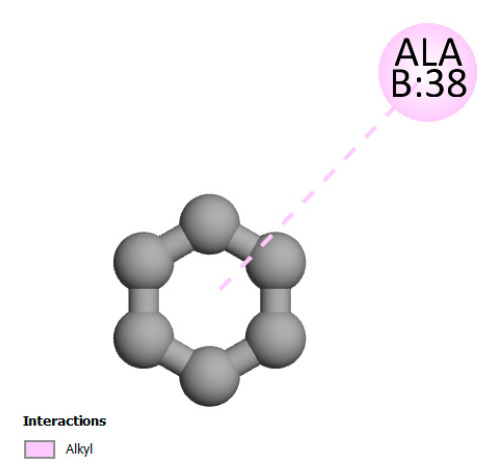	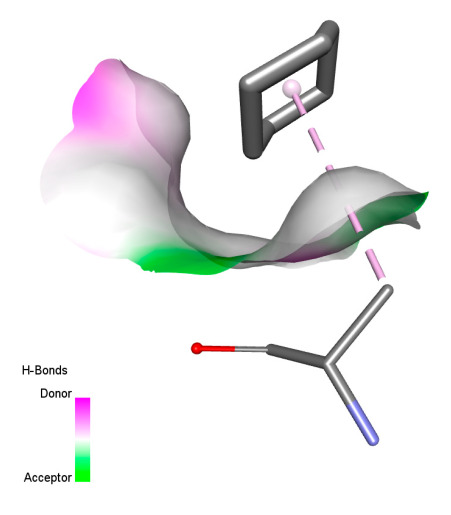
Quercetin-3-D-xyloside	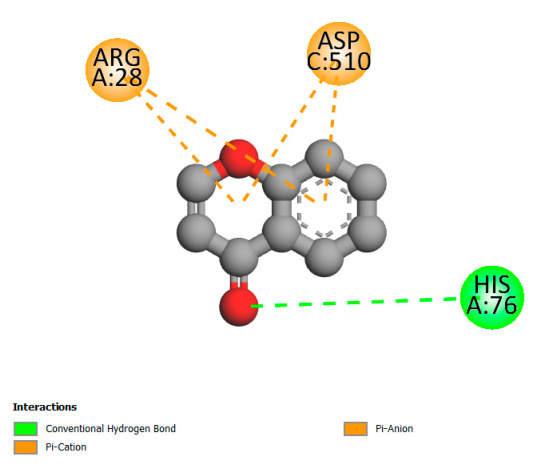	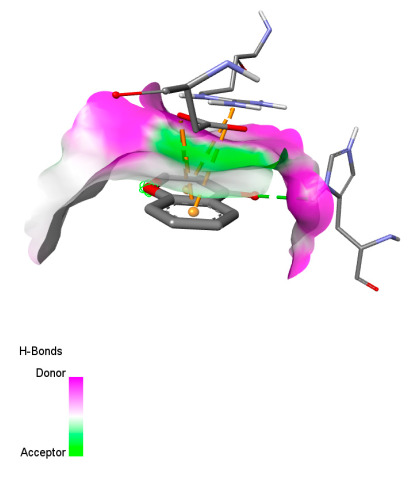	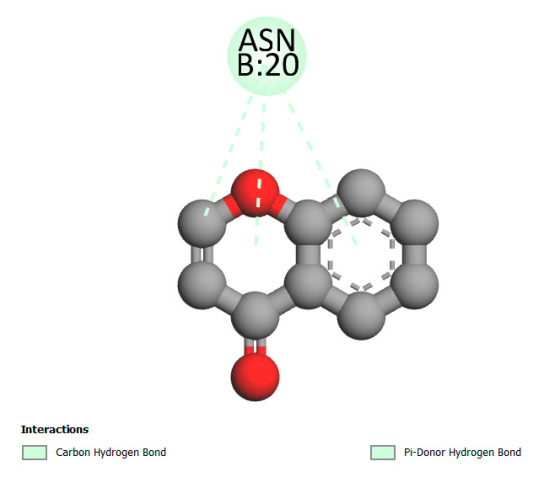	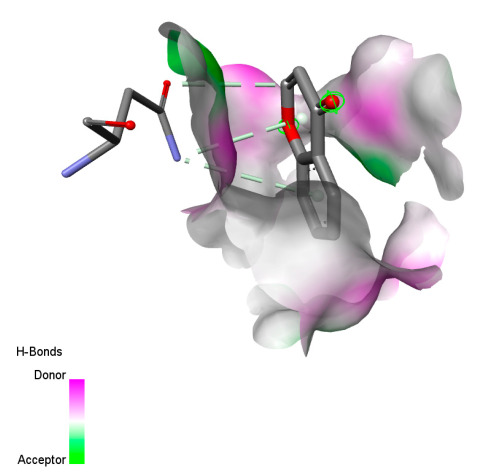
Quercetin-3-glucuronide	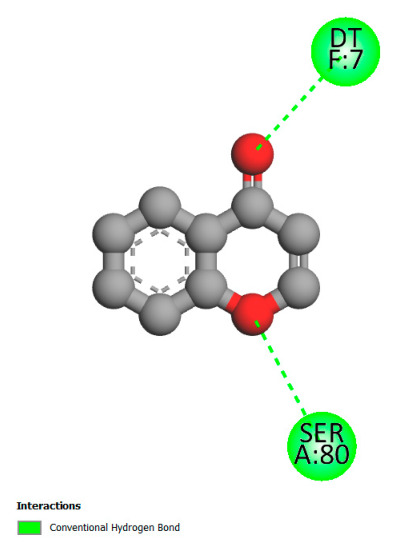	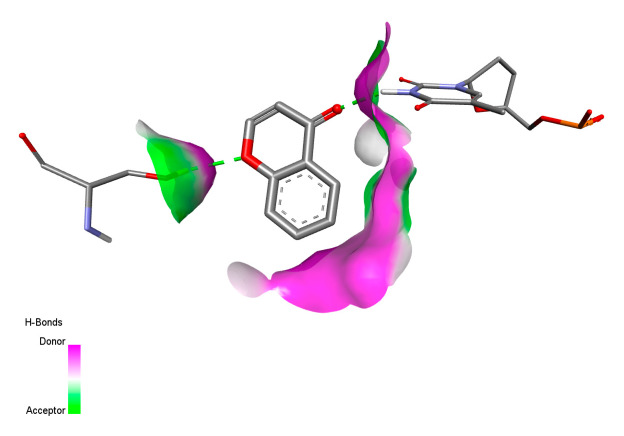	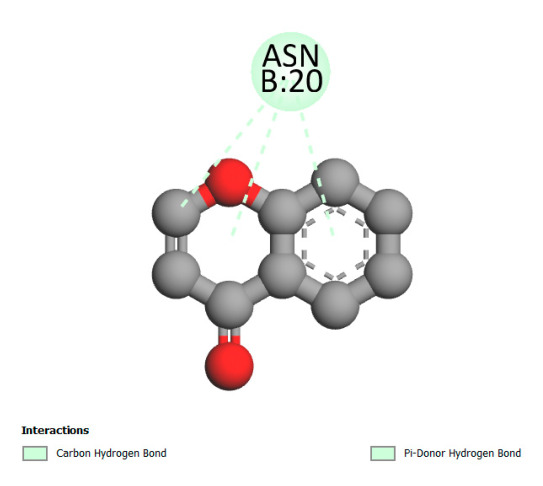	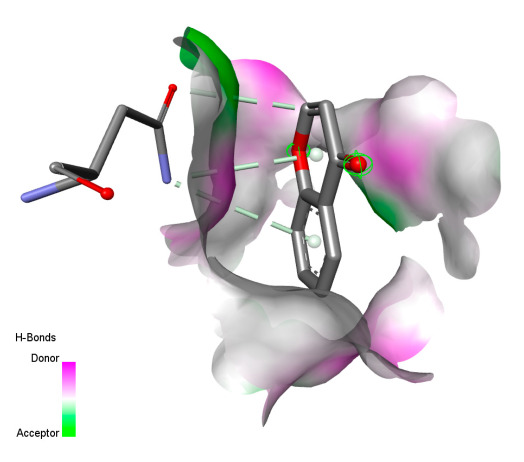
Rosmanol	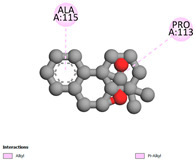	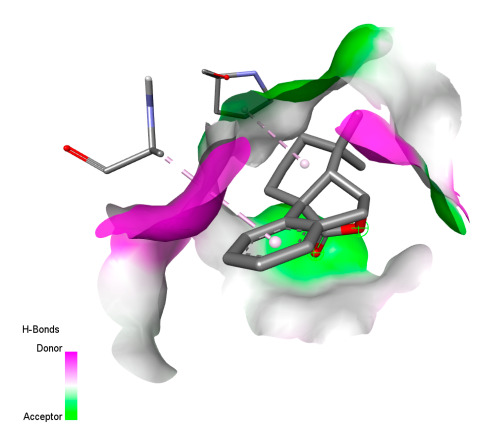	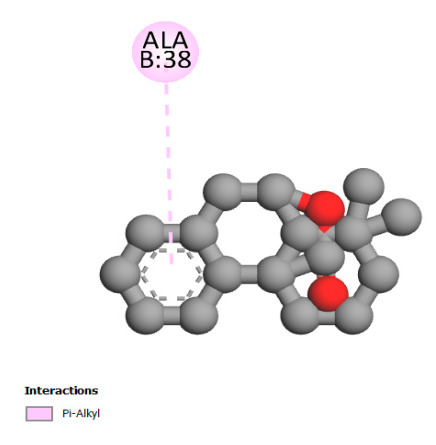	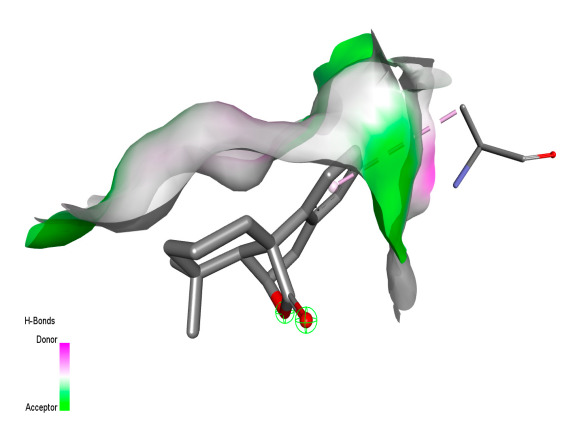
Salvianolic acid K	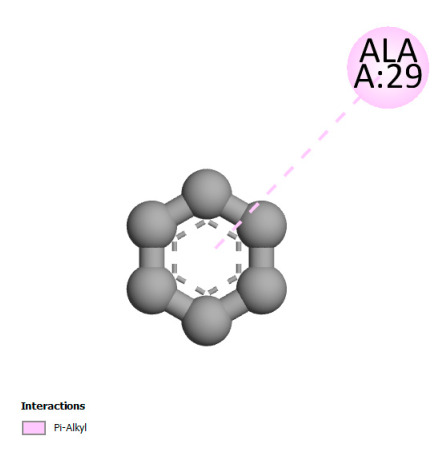	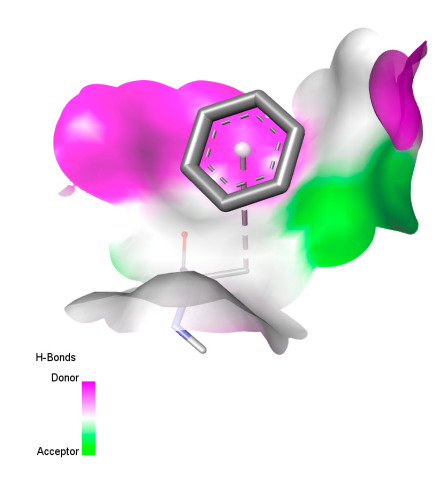	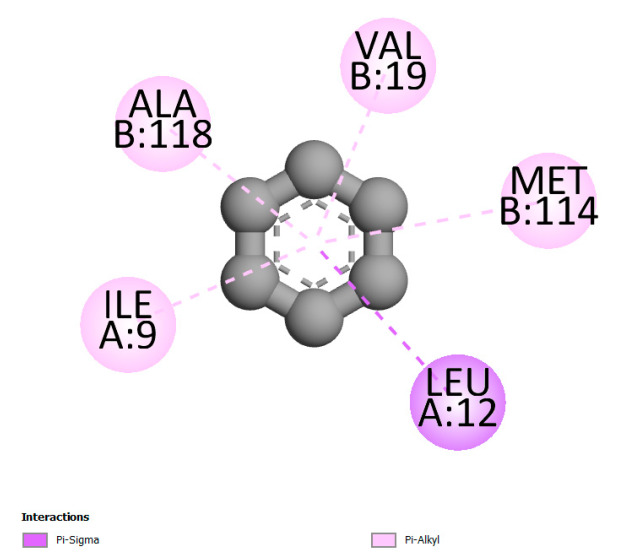	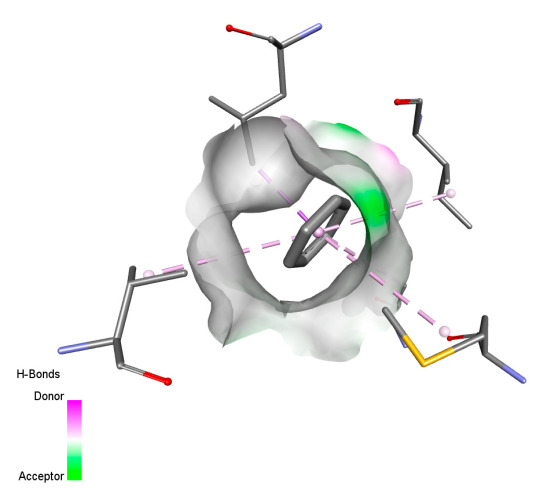

The antioxidant mechanism of *F. vulgare* extracts and essential oil primarily relies on their capacity to neutralize free radicals and to reduce transition metals, thereby preventing oxidative chain reactions. The predominant phenolic compounds found in the extracts, such as chlorogenic acid, quercetin-3-glucuronide, and dihydrocaffeic acid, have been identified as key contributors to this mechanism. These molecules act as electron or hydrogen atom donors, enabling them to stabilize free radicals, such as DPPH˙ and to convert ferric ions (Fe^3+^) into ferrous ions (Fe^2+^). Moreover, the significant correlation observed between the concentration of polyphenols and the outcomes of the DPPH, FRAP, and TAC assays underscores the pivotal role of these compounds in the antioxidant potential. This observation is consistent with the findings of Panda et al. (2019) [68], who demonstrated that the chemical modification of biomolecules like chitin by phenolic acids enhances both their water solubility and antioxidant activity. Additionally, oxygenated monoterpenes, such as fenchone and trans-anethole, although contributing to a lesser extent, further augment the overall effect by enhancing the scavenging of reactive oxygen species (ROS). These combined mechanisms highlight the potential of *F. vulgare* as a natural source of antioxidants capable of counteracting oxidative stress.

The comparative analysis reveals that phenolic extracts exhibit significantly higher antimicrobial and antioxidant activity than essential oils. This difference can be explained by the presence of several hydroxyl groups in polyphenols, which facilitate hydrogen and π-π interactions with the active sites of proteins, thereby enhancing their biological activity, as demonstrated by the research of Zhou et al. (2020) [69] and Chen et al. (2024) [70]. More specifically, the remarkable results observed for iso-chlorogenic acid A and salvianolic acid K, both derivatives of caffeic acid, highlight the importance of catechol motifs in modulating these biological activities, which has been confirmed by the work of Razaviamri et al. (2021) [71] and Dangles et al. (2012) [72]. In comparison, phenolic extracts outperform the monoterpenes found in essential oils, likely because they interact more effectively with target proteins.

However, although these results are promising and informative, further experimental validations are needed to confirm their effectiveness in real biological systems. This study paves the way for the therapeutic use of fennel, suggesting that the combination of phenolic extracts and essential oils in targeted formulations could lead to more effective therapeutic agents, a concept that aligns with the similar conclusions drawn by the research of Hosseini et al. (2021) [73].

The study of the antioxidant and antimicrobial activities of essential oils and fennel extracts reveals the richness of this plant in bioactive compounds that work synergistically to combat free radicals and pathogens. In vitro tests demonstrated that both the essential oil and extracts exhibit significant antioxidant capacities. Among the identified phenolic compounds, chlorogenic acid, quercetin-3-glucuronide, and dihydrocaffeic acid were found to be particularly effective in neutralizing free radicals, with IC50 values of 51.45 μg/mL for the essential oil and 93.71 μg/mL for the hydroethanolic extract. The antioxidant efficacy of the essential oil surpassed that of the aqueous extracts in the DPPH tests, confirming the superior potential of this extract form to combat oxidative stress, as noted in other similar studies [30,31].

Fennel extracts demonstrated notable antimicrobial activity, with particularly strong effectiveness against fungal pathogens, such as *Candida albicans* and *Aspergillus niger*, exhibiting minimum inhibitory concentrations (MIC) of 3.13 mg/mL and 6.25 mg/mL, respectively. Antimicrobial activity was also observed against bacteria, such as *Escherichia coli* and *Staphylococcus aureus*, highlighting a specificity of action depending on the type of microorganism. The in silico analyses further enriched these findings by identifying molecular mechanisms involving bioactive compounds, such as chlorogenic acid and quercetin-3-glucuronide. These compounds demonstrated strong affinities for antimicrobial target proteins, playing a central role in the observed activity. These findings open promising avenues for the development of natural therapeutic formulations and the exploration of synergies between active molecules.

Finally, although the main monoterpenes in the essential oil, such as fenchone (24.72%), trans-anethole (22.22%), and limonene (20.48%), play an important role, they contribute less to the antimicrobial activity than the phenolic extracts. These oxygenated monoterpenes, while displaying moderate antimicrobial action, enhance the overall effectiveness of the essential oil by complementing other components, in line with the findings of Alrub et al. (2023) [74]. This underscores the importance of combining different types of compounds found in fennel, where phenolic extracts dominate, but the monoterpenes also make a significant contribution to the overall biological activity.

## 3. Materials and Methods

### 3.1. Plant Material

**Figure 8:**
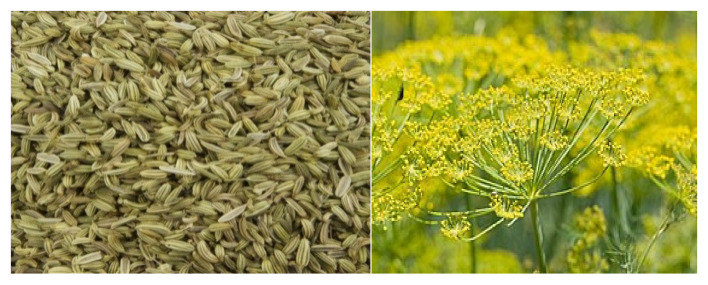
Morphological aspects of *Foeniculum vulgare* Mill.

*Foeniculum vulgare*, a member of the Apiaceae family, is a perennial herbaceous plant characterized by its yellow flowers and feathery, delicate foliage (Figure 8). The plant samples were collected from the Meknes region (Table 11). A summary of the species’ key identification criteria is provided in Table 12. The seeds were carefully dried in the shade and ground into a fine powder, which was subsequently used for the preparation of various extracts.

### 3.2. Quality Control of Plant Material

#### 3.2.1. Moisture Content (MC)

The water content of the plant material was determined as previously assayed by Saidi et al. (2023) [75]. In this method, 5 g of the plant was maintained in the oven at 105 °C for 24 h. The humidity level is calculated by the following Equation (1):

(1) $$MC\;\left(\%\right)=\frac{\left(m_{1}-m_{2}\right)}{m_{1}}\times 100$$

m_1_: initial mass of the plant before drying in the oven (g),

m_2_: final mass of the plant after drying in the oven (g).

#### 3.2.2. Determination of pH

The pH is determined after adding 10 mL of hot distilled water to 2 g of the plant. The STPURE double electrode was immersed in the filtrate of the mixture to note the pH value on an Ohaus Starter 3100 pH meter (Remok et al., 2023) [76].

#### 3.2.3. Determination of Titratable Acidity

The assay of titratable acidity, expressed as citric acid content per unit volume, is determined by titrimetry using a 0.01 N sodium hydroxide solution, in the presence of phenolphthalein as a colored indicator. Ten grams of plant powder was added to 50 mL of boiling distilled water, then the solution was stirred for 15 min. The mixture was adjusted to 100 mL with distilled water. After filtration, 10 mL of the filtrate to which 20 mL of distilled water were added, and were titrated with a solution of NaOH (0.01 N) with a few drops of phenolphthalein, until the color changed to pink. The noted titration volume was converted into citric acid equivalent following the Equation (2) below (Bergeron, 1995) [77]:

(2) $$Total\;Acidity=\frac{Dilution\;factor\times Weight\;of\;eq.\;Acide\times normality\;of\;NaOH\times titration\;vol.\;(mL)}{Sample\;mass\;(g)}$$

#### 3.2.4. Ash Content

The ash content was produced by incineration, according to the standard (1977). 2 g of the crushed plant material were placed in nickel crucibles, then puted into the muffle furnace at a temperature of 550 °C until all carbon particles were completely destroyed. The organic matter content is calculated by the following Formula (3):

(3) $$OM\%=\frac{m_{1}-m_{2}}{TS}\times 100$$

Bulleted lists look like this:

OM%: Organic matter;

m_1_: Pre-calcination capsule and sample mass;

m_2_: Post-calcination capsule and sample mass;

TS: Test sample.

The ash content was calculated as follows (4):

Ash%= 100 − MO% (4)

#### 3.2.5. Dosage of Metallic Trace Elements (MTE) by ICP-AES

The trace metals analysis (As, Cr, Sb, Pb, Cd, Fe, Cu and Ti) were carried out using the standardized mineralization protocol (AFNOR, 1985), using aqua regia reagent (HNO_3_ + 3HCl). One-tenth gram of plant material was mixed with 3 mL of aqua regia prepared from 1 mL of nitric acid HNO_3_ (99%) and 2 mL of hydrochloric acid HCl (37%). This mixture was placed in a reflux assembly at 200 °C for 2 h. After decantation, the supernatant was filtered through a 0.45 μm membrane. The filtrate was made up to 15 mL with distilled water. The concentrations of heavy metals were determined through ICP-AES analysis (Ultima 2 Jobin Yvon) at the UATRS laboratory (Technical Support Unit for Scientific Research) of the CNRST in Rabat [78].

### 3.3. Phytochemical Screening

The characterization tests for the different chemical groups are based on coloring, complexation and precipitation reactions, according to the methods described by Dohou et al., Judith, Mezzoug, et al., Bekro et al., Bruneton, and N’Guessan et al. [79,80,81,82,83,84].

#### 3.3.1. Primary Metabolites

The determination of primary metabolites in plants is crucial for understanding their physiological and biochemical processes. The extract’s interaction with iodized water allowed for the identification of the polysaccharide’s presence and type, while the Fehling method was applied to identify the reduced sugars. Two methods have been used to characterize the proteins: the reaction with biuret, which produces a colored complex (purple or mauve) when a few drops of copper sulfate are added to a base environment, and the reaction with xanthoproteins, which reveals the presence of specific aminated acids by heating nitric acid upon contact with the solution to be analyzed. The process of lipid detection involves adding an acid anhydride in an acidic environment to the extract to be analyzed, which turns red.

#### 3.3.2. Secondary Metabolites

Determining the secondary metabolites of plants is essential to comprehending their ecological, medicinal, and industrial functions. Several analytical techniques are used to identify and quantify these compounds. The precipitation of salts following the use of Mayer and Dargendorff’s reagent made it possible to highlight the presence of alkaloids. The tannins, or gallic tannins, have been identified using the Stiasny reaction, sodium acetate, and ferric chloride, while concentrated hydrochloric acid and isoamylic alcohol have been found to contain catechin tannins. The cyanidine reaction also revealed the leuco-anthocyanes, but without the addition of magnesium copeaux that were used to highlight the free flavonoids. Ten percent sulfuric acid and 25% NH_4_OH were added, which allowed the anthocyanes to be detected. A 25% dilution of ammoniac was used to highlight the anthracene derivatives. By using the right chemical products and potassium hydroxyde, cardiac glycosides have been discovered. The addition of strong sulfuric acid revealed the presence of sterols and triterpenes. Analyzing each sample’s mousse index revealed the presence of saponosides, which are distinguished by their ability to foam in aqueous solutions. One hundred percent ethanol was added to strengthen the aqueous extraction. After that, the watery extraction is enhanced with strong sulfuric acid and an ethanol solution infused with thymol to extract the oses and holosides.

### 3.4. Preparation of Seed Extracts of F. vulgare

#### 3.4.1. Extraction by Soxhlet

A sample of 30 g of powdered fennel seeds was placed in a cartridge inside the soxhlet extraction chamber. The plant material is extracted with 350 mL of solvent consisting of either a mixture of ethanol/water (70/30) E (1) or water alone E (0) (Table 13). Several cycles are necessary to exhaust the plant material. After filtration, the solvent was evaporated to dryness under reduced pressure at 50 °C using a rotary evaporator. Then the extracts were stored at 4 °C until they are used for further analyses [85].

#### 3.4.2. Extraction by Decoction

The preparation of the aqueous extract (E (2)) was carried out by the seeds of the plant dried and pulverized into a fine powder (Table 13). Then, 30 g of the crushed plant were added to 350 mL of distilled water, then this mixture was introduced into a reflux assembly, and heated to 80 °C with stirring for one hour, then filtered. The extract was dried in an oven at 70 °C for 18 h then placed in amber glass vials [85].

### 3.5. Dosage of Phenolic Compounds

#### 3.5.1. Determination of Total Polyphenols

The contents of total phenolic compounds were determined by the Folin–Ciocalteu colorimetric method described by Singleton and Rossi [86]. The reaction mixture is composed of 20 µL of plant extract studied, 1.5 mL of a sodium carbonate solution (75 g/L) and 1.5 mL of Folin–Ciocalteu reagent (phosphomolybdic H_3_PMO_12_O_40_) at 10% (*V*/*V*). Then, the tubes were incubated at room temperature and protected from light for 2 h. The absorbance was measured at 760 nm and the results are expressed in milligrams of gallic acid equivalent per gram of dry matter (mg EAG/g of plant).

#### 3.5.2. Dosage of Flavonoids

The determination of the total flavonoid content of plant extracts was carried out by the aluminum chloride (AlCl_3_) method. Indeed, 2 mL of distilled water and 10 µL of aluminum chloride prepared in methanol (10% m/V) were added. The mixture was supplemented with absolute methanol up to a total volume of 5 mL. The solutions were homogenized and then left for 2 h in the dark. The absorbance was determined at 433 nm. Flavonoid contents were expressed as quercetin equivalents (QE) [87,88].

#### 3.5.3. Dosage of Condensed Tannins

The determination of condensed tannins was carried out spectrophotometrically with vanillin in an acidic methanolic medium according to Price et al. (1978) [89]. The reaction mixture was composed of one volume of extract, 3 mL of methanolic vanillin solution (4%, m/V) and 1.5 mL of HCl (37%). This mixture was shaken then incubated at room temperature and in the dark for 20 min. The absorbance was measured at 499 nm and the concentration of condensed tannins was expressed as milligram equivalent to catechin per gram of extract (mg CE/g).

### 3.6. Identification of Chemical Composition by HPLC/UV-DAD

The analysis of the chemical composition of the extracts of the plants studied was carried out by HPLC-UV-DAD (Thermofisher Scientific Ultimate 3000, Sunnyvale, CA, USA). A volume of 10 µL of each extract was dissolved in distilled water to obtain a concentration of 100 µg/mL, filtered on PTFE syringe filters with 0.20 µm pore size (Interchim^®^, Montluçon, France), then injected into a C18 column 100 mm long, 2.1 mm in diameter and whose pores have a diameter of 1.7 µm. The temperature was set at 30 °C and the flow rate at 0.45 mL/min. The mobile phase was composed of two solvents: solvent A (Water + formic acid (0.1%), *v*/*v*) and solvent B (Acetonitrile + formic acid (0.1%), *v*/*v*). The elution gradient established was A + B [98:2] (0–19 min), A + B [70:30] (20–24 min), A + B [5:95] (25 min) and A + B [98:2] (26–30 min). Detection was carried out using a diode array detector at wavelengths of 280 nm, 320 nm and 360 nm. The standards used are chlorogenic acid, quinic acid, quercetin glucuronide, rosmarinate methyl, Syringic acid hexoside, luteolin, Kaempferol, diosmetin, Ascorbyl monomyristate pinoresinol, Galloylquinic acid, catechin obtained from Sigma-Aldrich^®^, Allentown, PA, USA, cinnamic acid (Rhône-Poulenc, Lyon, France), rosmarinic acid (Extrasynthesis, Genay, France), gallic acid (Prolabo, Paris, France), protocatechuic acid (Koch-Light Laboratories LTD, London, UK), apigenin (Carl Roth, Karlsruhe, Germany), coumarin (HPLC grade, Sigma-Aldrich, Behringer, Germany), luteolin, myricetin, caffeic acid and ferulic acid (Sigma, USA), apigetrin, vanillic acid, naringenin7-O-glucoside, and 3-Feruloylquinic acid (Merck, Darmstadt, Germany). The compounds were characterized solely by matching the retention times and UV spectra of the obtained peaks [90].

### 3.7. Extraction and Determination of Essential Oil Yield

The dried fennel seeds were subjected to hydrodistillation for 3 h using a Clevenger type apparatus. The essential oil obtained was dried over anhydrous sodium sulfate, filtered and stored at −4 °C until tested and analyzed. The essential oil (EO) yield is expressed in mL/100 g of dry matter [91].

#### 3.7.1. Density

The density of an EO at 20 °C is the ratio between the density of this oil and the density of water at the same temperature. It was determined according to the following formula [92].

(5) $$d_{20\;}=\frac{m_{2}-m_{0}}{m_{1}-m_{0}}$$

With:

m_0_(g): Mass of the empty pycnometer;

m_1_(g): Mass of the pycnometer filled with water;

m_2_(g): Mass of the pycnometer filled with oil.

#### 3.7.2. Analysis of Essential Oil by Gas Chromatography Mass Spectrometry

The EO were analyzed chromatographically using a Thermo Electron Trace GC Ultra gas chromatograph in conjunction with a Thermo Electron Trace MS mass spectrometer (Thermo Electron: Trace GC Ultra; Polaris Q MS, Rodano, Italy). Fragmentation occurred via electron impact at an intensity of 70 eV. The chromatograph utilized a DB-5 column (5% phenyl-methyl-siloxane, 30 m × 0.25 mm × 0.25 μm film thickness) in conjunction with a flame ionization detector (FID) operated by a H_2_/Air gas mixture. The column temperature was set to rise at a rate of 4 °C per minute, beginning at 50 °C and reaching 200 °C, followed by a 5-min hold period. The injection mode was configured to split, with a split ratio of 1:70 and a flow rate of 1 mL/min. Nitrogen was utilized as the carrier gas, also at a flow rate of 1 mL/min. The essential oils’ chemical composition was determined by comparing their calculated Kovats indices (IK) with those documented by Adams and other reference products in the literature [93,94,95]. Furthermore, retention indices and mass spectra were compared with data from the National Institute of Standards and Technology (NIST) mass spectral libraries. Experimental retention indices were compared with those found in the NIST online database (https://webbook.nist.gov/chemistry/name-ser/, accessed on 23 December 2024). The individual component proportions were automatically calculated from the total ion count identified by the GC-MS and presented as percentage compositions.

### 3.8. Antimicrobial Activity

#### 3.8.1. Microbial Material

To determine the antimicrobial activity of the aqueous extract of the plants studied, we chose five bacterial strains common in human pathologies, belonging to Gram-positive and Gram-negative bacteria and five fungal strains (yeast and fungi) (Table 14).

#### 3.8.2. Determination of Minimum Inhibitory Concentration, Minimum Bactericidal Concentration, and Minimum Fungicidal Concentration

The Minimum Inhibitory Concentration (MIC) is defined as the lowest concentration of an essential oil (EO) or extract that effectively prevents the growth of a microorganism. The MIC was assessed utilizing the microdilution method [96]. A stock solution of the essential oil, formulated in 10% DMSO, was serially diluted to obtain concentrations from 5 to 0.93 × 10^−2^ mg/mL for the essential oil. A stock solution was prepared for the extracts and subsequently diluted to the required concentrations, expressed in mg/mL. Dilutions of essential oils and extracts were prepared in Mueller–Hinton broth for bacterial cultures and Sabouraud broth for fungal cultures, achieving a final volume of 100 µL per concentration. Subsequently, 100 µL of the microbial inoculum, calibrated to a final concentration of 10⁶ CFU/mL for bacteria or 10⁴ CFU/mL for fungi, was incorporated into the dilution series. Following 24 h of incubation at 37 °C, 10 µL of resazurin was introduced to each well to indicate microbial growth. After a further 2 h incubation at 37 °C, microbial growth was evidenced by a color transition from violet to pink. The MIC was identified as the minimal concentration that inhibited this color alteration. The 11th and 12th wells of each series functioned as the growth control and sterility control, respectively. The method was conducted twice for both the essential oil and the extracts. To ascertain the Minimum Bactericidal Concentration (MBC) or Minimum Fungicidal Concentration (MFC), 10 µL was extracted from each well exhibiting no apparent growth and put into Mueller–Hinton agar (MH) for bacterial assessment or Sabouraud agar for fungal evaluation. The plates were incubated for 24 h at 37 degrees Celsius. The MBC or MFC was established as the minimal concentration of the sample that achieved a 99.99% decrease in CFU/mL relative to the control. The MBC/MIC or MFC/MIC ratio was computed to assess the antibacterial efficacy. A ratio below 4 signifies a bactericidal/fungicidal impact, whilst a ratio beyond 4 indicates a bacteriostatic/fungistatic effect of the material.

### 3.9. Antioxidant Activity

#### 3.9.1. DPPH* Trapping Free Radicals

The antioxidant activity of the essential oil and various seed extracts were evaluated to measure their scavenging capacities for stable 2,2′-diphenyl-1-picrylhydrazyl radicals. The DPPH test was performed as described [97]. The test is carried out by mixing a volume of 2.8 mL of the previous solution of DPPH˙ with 200 µL (0.004% methanolic solution of DPPH) of each sample to be tested or of standard antioxidant (ascorbic acid) at different concentrations (from 0 to 200 µg/mL) in methanol. At the same time, a negative control is prepared by mixing 200 μL of ethanol with 2.8 mL of the ethanol solution of DPPH*. After 30 min of incubation at room temperature in the dark. The absorbance reading is taken against a blank at 517 nm. The results obtained were expressed as percentage inhibition (PI%):

(6) $$PI\left(\%\right)=\frac{A_{Control}-A_{test}}{A_{control}\;}\;*\;100$$

With:

A_control_: Absorbance of the solution containing only the solution of the DPPH radical;

A_sample_: Absorbance of the solution of the samples to be tested in the presence of DPPH.

IC_50_ or 50% Inhibitory Concentration is the concentration of the test sample necessary to reduce 50% of the DPPH* radical. The IC_50_ are calculated graphically by linear regressions.

#### 3.9.2. FRAP Iron Reduction Power Test

The ability of phenolic extracts from C. sativum to reduce ferric iron (Fe^3+^) to ferrous iron (Fe^2+^) in potassium ferricyanide was assessed using the method outlined by Oyaizu [98]. In this assay, 1 mL of the plant extract was combined with 2.5 mL of phosphate buffer (0.2 M, pH 6.6) and 2.5 mL of a 1% potassium ferricyanide solution (K_3_Fe(CN)_6_). The mixture underwent incubation in a water bath at 50 °C for 20 min, followed by the addition of 2.5 mL of 10% trichloroacetic acid to terminate the reaction. The solution was centrifuged at 3000 rpm for a duration of 10 min. Following this, 2.5 mL of the supernatant was mixed with 2.5 mL of distilled water and 0.5 mL of a 0.1% aqueous FeCl_3_ solution. The absorbance of the reaction medium was measured at 700 nm using a UV-Vis spectrophotometer, with a blank prepared by replacing the extract with distilled water for calibration. Butylated Hydroxyanisole (BHA) was used as the positive control, and its absorbance was measured under identical conditions. All experiments were performed in triplicate. The reducing power was plotted as a function of BHT concentration or the extract concentration, and the EC_50_ value, corresponding to an absorbance of 0.5, was determined from the graph.

#### 3.9.3. Total Antioxidant Capacity (TAC)

The Total Antioxidant Capacity (TAC) of the plant extracts was assessed using the phosphomolybdenum method, as described by Khiya [99]. This technique is based on the reduction in molybdenum (VI), present as molybdate ions (MoO_4_^2−^), to molybdenum (V) (MoO_2_^+^) in the presence of antioxidant compounds, leading to the formation of a green phosphate/Mo(V) complex under acidic conditions. For the assay, 3 mL of the reagent solution was mixed with 0.3 mL of the extract, with the reagent solution composed of 0.6 M sulfuric acid, 28 mM sodium phosphate, and 4 mM ammonium molybdate. The reaction mixtures were then sealed and incubated at 95 °C for 90 min to ensure complete reduction. After cooling to room temperature, the absorbance of the resulting solutions was recorded at 695 nm using a UV-Vis spectrophotometer, with a blank sample prepared under identical conditions serving as the reference. To quantify the antioxidant capacity, several concentrations of ascorbic acid were used to construct a standard calibration curve. The results were expressed as milligrams of ascorbic acid equivalents per gram of crude extract (mg AAE/g), providing a comparative measure of the extracts’ overall antioxidant potential.

### 3.10. Molecular Docking

The three-dimensional structures of the target proteins listed in Table 15 were obtained from the RCSB Protein Data Bank (https://www.rcsb.org/, accessed on 23 December 2024) and subsequently visualized using the UCSF Chimera software (Chimera-1.17). This preliminary step was crucial for facilitating the subsequent molecular docking analyses. The protein structures underwent a rigorous preprocessing protocol, leveraging the combined functionalities of Chimera and AutoDock Tools (version 1.5.6, The Scripps Research Institute, La Jolla, CA, USA). This protocol involved the removal of water molecules, heteroatoms, non-essential protein chains, and co-crystallized ligands, with the aim of optimizing the accessibility and specificity of the active site. To ensure an accurate representation of molecular interactions, polar hydrogen atoms were added, and Gasteiger partial charges were assigned. The processed protein structures were then converted into the pdbqt format, required for docking simulations using AutoDock Vina 1.2.0 (accessed on 23 December 2024). As for the ligands, three-dimensional models of the eighteen compounds under investigation were retrieved from the PubChem database and subjected to energy minimization to ensure conformational stability. These minimized structures were converted into the pdbqt format using OpenBabel (http://openbabel.org/; accessed on 23 December 2024), thereby ensuring compatibility with the docking engine. Molecular docking simulations were performed using AutoDock Vina, which employs an empirical scoring function to estimate the binding affinities between ligands and their target proteins. For each protein, a three-dimensional grid was defined around the active site, with meticulously adjusted coordinates and dimensions to encompass the targeted binding pocket. Post-docking analysis was carried out using PyMOL version 2.5.5 (accessed on 23 December 2024), enabling detailed visualization of molecular interactions (such as hydrogen bonds and hydrophobic contacts) and the assessment of associated binding free energies. To validate the reliability of the docking protocol, a re-docking procedure was implemented as follows: the co-crystallized ligand was removed and subsequently re-docked into the original binding region. The spatial overlap between the docked and crystallographic conformations was evaluated using the Root Mean Square Deviation (RMSD) value. An RMSD value below 2 Å was considered indicative of the structural and statistical validity of the docking methodology employed.

### 3.11. Statistical Analysis

The data were expressed as mean ± standard error of the mean (SEM). The threshold for statistical significance was set at *p* < 0.05. Statistical analyses were carried out using a one-way analysis of variance (ANOVA), followed by a Tukey–Kramer multiple comparisons post hoc test, in order to determine significant differences between groups. All analyses were performed using GraphPad Prism version 8.0 for Windows (GraphPad Software Inc., San Diego, CA, USA).

## 4. Conclusions

This study demonstrates the remarkable therapeutic potential of *F. vulgare*, whose extracts and essential oil are characterized by a composition rich in bioactive compounds, notably, phenolic acids (chlorogenic acid, 14.79%), flavonoids (quercetin-3-glucuronide), and terpenes (fenchone 24.72%, trans-anethole 22.22%). The analyses revealed significant antioxidant activity, particularly pronounced in the essential oil (IC_50_ = 51.45 µg/mL) and the hydroethanolic extract, which is correlated with their high polyphenol content. Regarding antimicrobial activity, notable fungicidal action was observed against *Candida albicans* (MIC = 3.13 mg/mL) and *Aspergillus niger* (6.25 mg/mL), attributed to the predominant monoterpenes. In silico approaches confirmed the preferred interactions of these compounds with key enzymatic targets involved in oxidative stress and microbial proteases, thus validating the proposed mechanisms of action. These results position *F. vulgare* as a promising resource for the development of natural therapeutic agents, although preclinical and clinical studies remain necessary to optimize its applications in human health. The complementarity of phenolic extracts and essential oil particularly opens interesting perspectives for synergistic formulations in pharmacology and nutraceuticals. Thus, to ensure the safe application of *F. vulgare* extracts, it is imperative to elucidate their toxicological profile. In the absence of such data, in vitro cytotoxicity assays or comprehensive safety assessments should be considered as essential steps to guide future research directions.

## Figures and Tables

**Table 1:** Quality control of plant matter, including moisture content (MC), pH, acidity, organic material (OM), and mineral matter (MM).

Species	MC (%)	pH	Acidity	MM (%)	MO (%)
*F. vulgare*	25.12 ± 0.001	5.5 ± 0.00	0.11 ± 0.00	6.4 ± 0.067	93.6

**Table 2:** Concentration of heavy metals (mg/L) (ICP) and FAO/WHO Maximum Limit (2009).

Species	Arsenic (As)	Chrome (Cr)	Antimoine (Sb)	Plomb (Pb)	Cadmium (Cd)	Iron (Fe)	Copper (Cu)	Titanium (Ti)
***F. vulgare***	0.0058	0.0008	0.0023	Undetectable	Undetectable	0.271	0.003	Undetectable
**Maximum Limit** (mg/L)	0.05	0.05	0.005	0.05	0.005	20	1	-

**Table 3:** Chemical families present in *F. vulgare*.

Chemical Group			*F. vulgare*
**Secondary Metabolites**	Lipids (Lieberman–Burchard reaction)		++
	Protein	Biuret reaction	+
		Xanthoprotein reaction	++
	Reducing sugar		+
	Polysaccharide		+
**Secondary Metabolites**	Tannins		+++
	Catechic tannins		+++
	Gallic tannins		+
	Flavonoids		++
	Cyanidin reaction		Flavones
	Leucoanthocyanins		++
	Saponosides		+
	Alkaloids		+
	Reducing compounds		++
	Monosaccharides and holosides		++
	Mucilages		++
	Sterols and triterpenes		++

+: weak positive test; ++: positive test; +++: strongly positive test.

**Table 4:** Percentages of the compounds classes identified in the *F. vulgare*.

Classes	R.A (%)		
	E (0)	E (1)	E (2)
Carboxylic ester	1.04	0	0.66
Dipeptide	0	0	5.42
Ester	0	0.55	0
Lignan	0	1.21	7.82
Phenolic compound	7.12	3.54	2.36
Fatty acid	0	0	1.67
Phenolic acid	49.96	33.39	37.3
Flavonoid	24.4	42.91	19.72
Phenolic diterpene	0	1.29	8.08
Polyphenol	11.2	9.94	7.84
Quinone	0	0	0.49
Terpenoid	4.42	6.58	6.46
Vitamin	1.82	0.56	1.84

**Table 5:** Yield and density of *F. vulgare* oils.

Species	Yield (%)	Density (g/mL)
***F. vulgare* Mill**	2.500 ± 0.067	0.964 ± 0.002

**Table 6:** Relative percentage composition of *F. vulgare* EO by GC-MS analysis.

Compound		RA %		KI	
α–Pinene		1.1		939	
Camphene		0.08		954	
Sabinene		0.1		975	
β–Pinene		0.03		979	
Myrcene		0.82		990	
α–Phellandrene		0.13		1002	
β–Phellandrene		0.05		1029	
Limonene		20.48		1029	
1,8-Cineole		0.11		1031	
β–cis-Ocimene		0.11		1037	
γ–Terpinene		0.85		1059	
Fenchone		24.72		1086	
Terpinolene		0.13		1088	
Cis-Thujone		0.03		1102	
Trans-Pinene hydrate		0.05		1122	
Camphor		0.52		1146	
Terpinen-4-ol		0.06		1177	
Methyl chavicol (estragole)		8.79		1196	
Fenchyl acetate <endo->		0.07		1220	
Fenchyl acetate <exo->		0.18		1232	
Cis-anethole		19.18		1252	
Trans-anethole		22.22		1284	
Anisyl methyl ketone		0.06		1382	
Germacrene D		0.07		1481	
Trans-Methyl isoeugenol		0.05		1492	
Identified compounds (%)			99.99		
Monoterpenes (%)			22.90		
Oxygenated monoterpenes (%)			77.02		
Sesquiterpenes (%)			0.07		
Oxygenated sesquiterpenes (%)			0.0		

RA: Relative abundance (%); KI: Kovats Index.

**Table 7:** Antimicrobial activity of *F. vulgare* EO and extracts.

Strains	EO (mg/mL)		Extracts (mg/mL)						Gentamicin	Terbinafin
			E (0)		E (1)		E (2)			
	MIC	MBC/MFC	MIC	MBC/MFC	MIC	MBC/MFC	MIC	MBC/MFC	MIC (µg/mL)	MIC (µg/mL)
*Enterobacter cloacae*	25	25	50	50	12.5	25	50	50	>4	-
*Klebsiella pneumoniae*	25	50	>50	>50	25	25	>50	>50	<=1	-
*Escherichia coli* sauvage	25	25	>50	>50	50	50	>50	>50	2	-
*Staphylococcus aureus* BLACT	50	50	50	50	50	50	50	50	<0.5	-
*Staphylococcus epidermidis*	50	100	50	50	50	50	>50	>50	2	-
*Candida albicans*	3.13	6.25	50	50	50	50	50	50	-	12.500
*Candida dubliniensis*	25	25	50	50	50	50	>50	>50	-	3.125
*Candida tropicalis*	12.5	25	50	50	12.5	12.5	>50	>50	-	12.500
*Candida parapsilosis*	25	50	50	50	0.78	0.78	>50	>50	-	6.250
*Aspergillus niger*	6.25	6.25	50	50	25	25	>50	>50	-	3.125

MIC: minimum inhibitory concentration; MBC: minimum bactericidal concentration; MFC: minimum fungicidal concentration.

**Table 8:** Docking scores of EO and extract compounds from *F. vulgare* against various target proteins (binding energies in kcal/mol).

Molecules\Proteins		Antimicrobial Activities										**Antioxidant Activities**				
		7TI1	3RAE	4DUH	2W9S	1JIJ	3KP5	7RJB	5V5Z	4YBF	4ZA5	**5qj2**	**3nrz**	**1og5**	**1n8q**	**2cdu**
Extracts	3-O-Caffeoylshikimic acid	−7.4	−8.4	−6.4	−7.9	−7.4	−8.5	−8.5	−7.6	−7.1	−7.9	−8.4	−8.6	−8.7	−8.8	−8.3
	7-Methoxy-2-methylisoflavone	−6.9	−7.9	−6	−7	−6.9	−7.7	−9.5	−7.3	−6.9	−8.2	−7.9	−8.1	−8.5	−8.6	−8.2
	Apigenin	−7	−8.4	−6.6	−8.1	−7	−8.2	−8.6	−7.2	−7	−8.4	−8.2	−9.1	−8.8	−9.1	−7.9
	Butyl ferulate	−6.2	−6.4	−4.8	−6.1	−6.2	−6.4	−7.1	−6.1	−5.9	−6.8	−6.1	−7.2	−6.8	−7.3	−7.5
	Chlorogenic acid	−7.2	−9	−6.5	−7.9	−7.2	−8.5	−8.5	−7.6	−7.2	−8.4	−8	−8.9	−8.8	−8.7	−8.2
	Dihydrocaffeic acid	−5.9	−6.2	−5.1	−6.6	−5.9	−6.3	−6.3	−5.8	−5.7	−6.3	−6	−7.2	−6.3	−6.6	−6.4
	Embelic acid	−5.5	−6.4	−4.6	−6.3	−5.5	−7	−6.9	−6.3	−5.4	−6.7	−6.3	−7.3	−6.9	−7.6	−7.3
	Isochlorogenic acid A	−8.1	−9.3	−8.2	−8.6	−8.1	−10.3	−9.7	−8.5	−8.6	−9.2	−8.7	−9.9	−9.1	−9	−8.3
	Medioresinol	−7.2	−8.6	−6.5	−7.9	−7.2	−8.9	−8.3	−7.6	−7.2	−7.9	−9	−7.8	−7.8	−8.4	−8.7
	Quercetin-3-D-xyloside	−7.6	−9.9	−7.1	−8	−7.6	−8.5	−9.1	−7.6	−7.7	−8.5	−9.2	−8.1	−8.8	−9.8	−8.1
	Quercetin-3-glucuronide	−7.6	−9.3	−7.1	−8	−7.6	−8.5	−9.1	−7.6	−7.7	−8.6	−9.1	−8.1	−8.8	−9.1	−8.1
	Rosmanol	−7.8	−9.9	−7.2	−7.5	−7.8	−6.8	−8.3	−7.7	−7.8	−8.2	−9.1	−7.9	−9.4	−8.4	−8.6
	Salvianolic acid K	−8	−9.4	−7.3	−9	−8	−9.5	−8.7	−8.6	−8	−9.5	−9	−10.9	−10	−9.6	−7.9
EO	Fenchone	−5.6	−5.1	−4.8	−5.5	−5.6	−6.5	−5.6	−5.7	−5.4	−6	−5.4	−5.7	−5.9	−6.1	−6.3
	Trans-anethole	−5.7	−5.1	−4.3	−5.2	−5.7	−5.7	−6.8	−5.3	−5	−5.6	−5	−6.5	−6	−6.1	−6.4
	Limonene	−5.8	−5	−4.3	−5	−5.8	−6.1	−6.9	−5.5	−5.1	−5.8	−5.4	−6.5	−5.8	−6	−6.5
	Cis-anethole	−5.7	−5	−4.2	−5.2	−5.7	−6.1	−6.6	−5.7	−4.9	−5.5	−5	−6.3	−5.8	−5.9	−6.5
	Estragole	−5.8	−5.2	−4.2	−5.2	−5.8	−5.9	−6.6	−5.4	−4.8	−5.6	−5.2	−6.2	−5.7	−6	−6.1

**Table 10:** Two-dimensional and three-dimensional interactions of *F. vulgare* compounds with target proteins involved in antioxidant activities.

Molecules\Proteins	3NRZ		1OG5	
	2D	3D	2D	**3D**
Apigenin	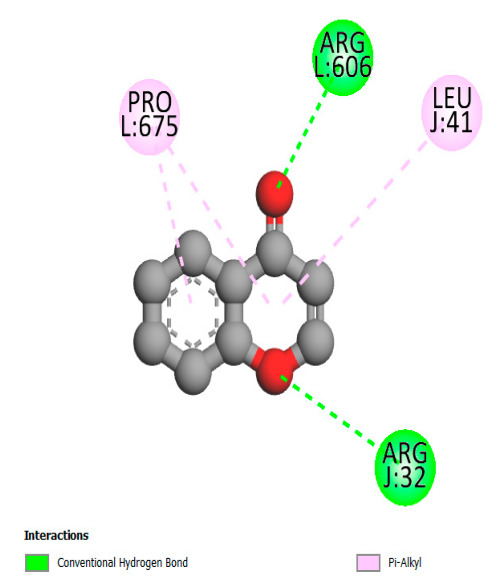	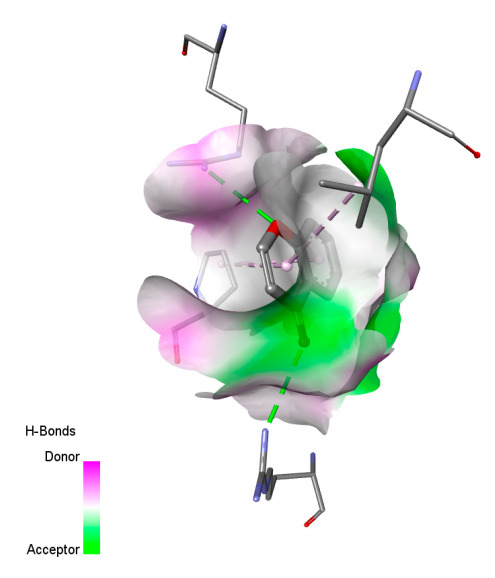	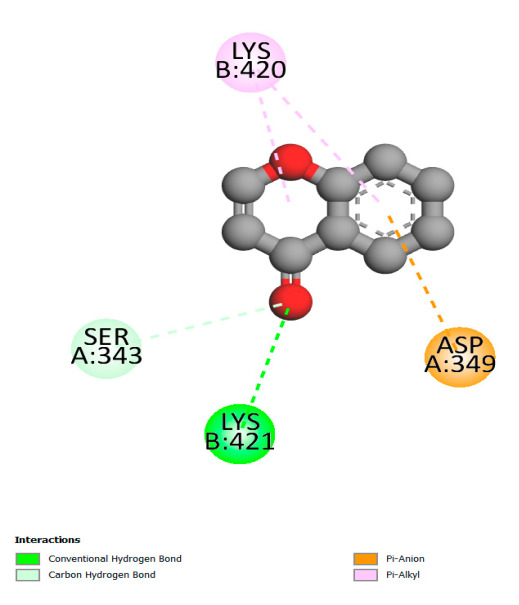	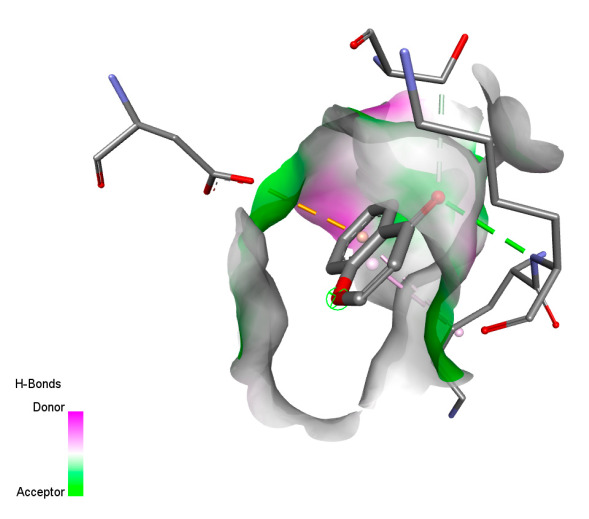
Chlorogenic acid	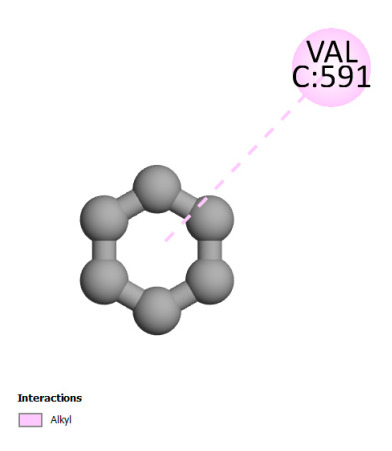	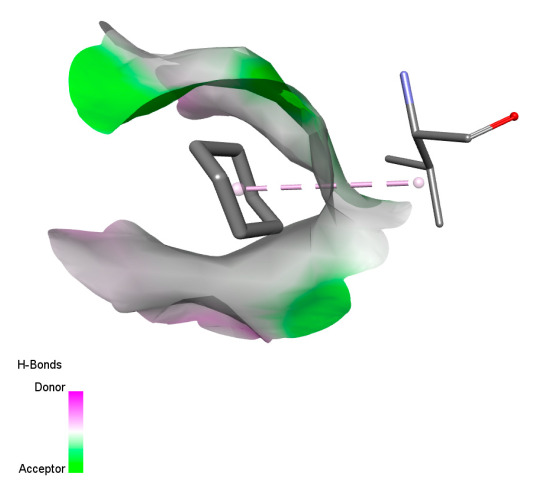	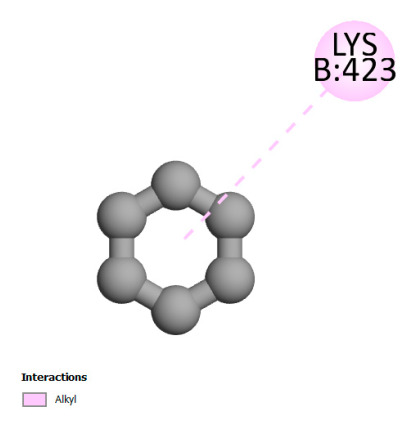	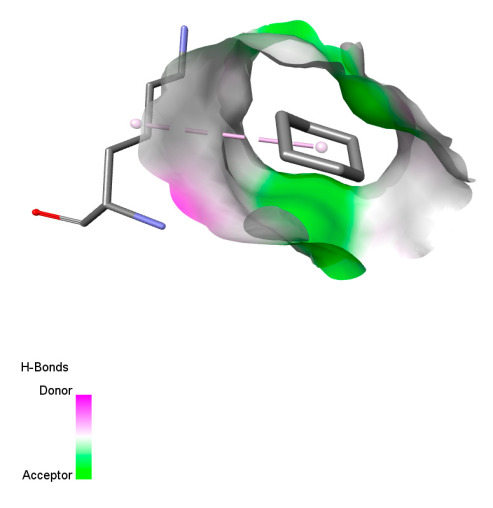
Isochlorogenic acid A	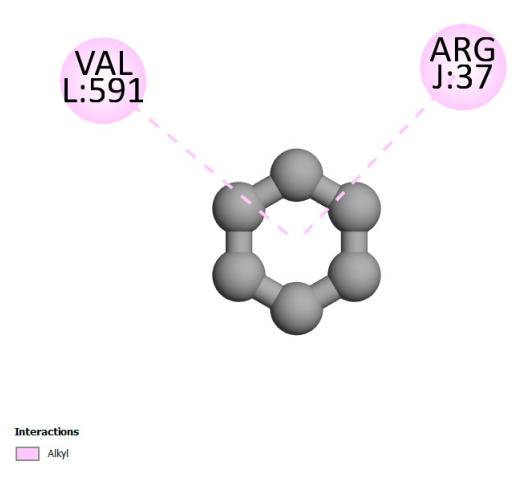	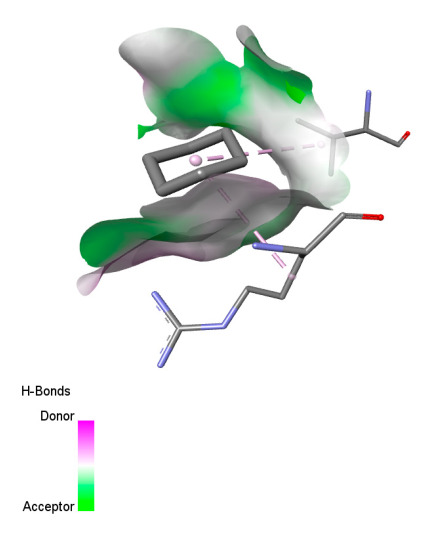	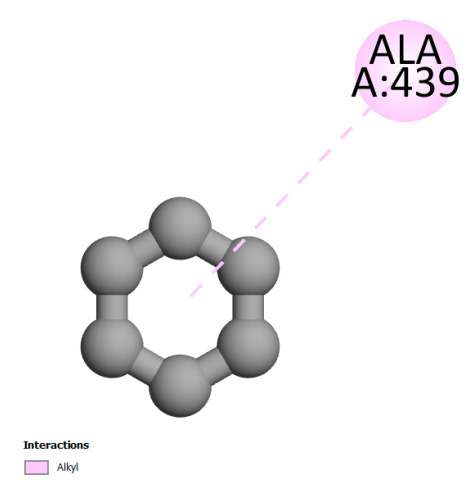	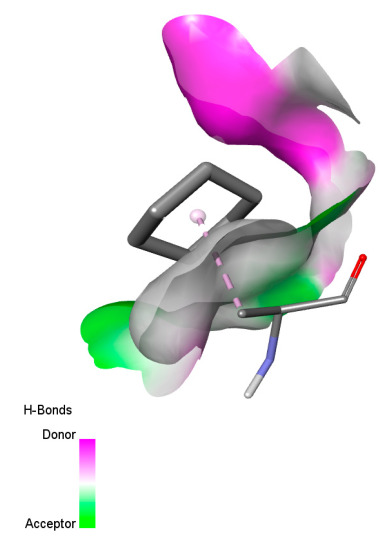
Rosmanol	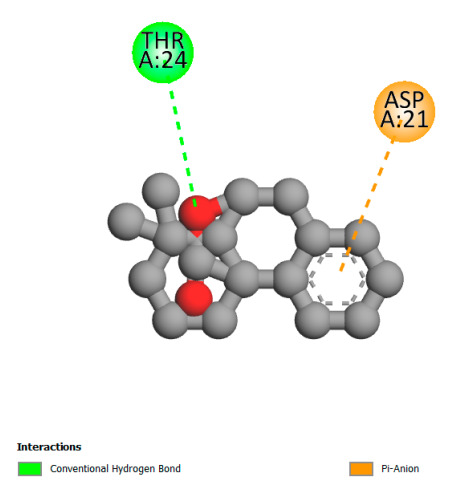	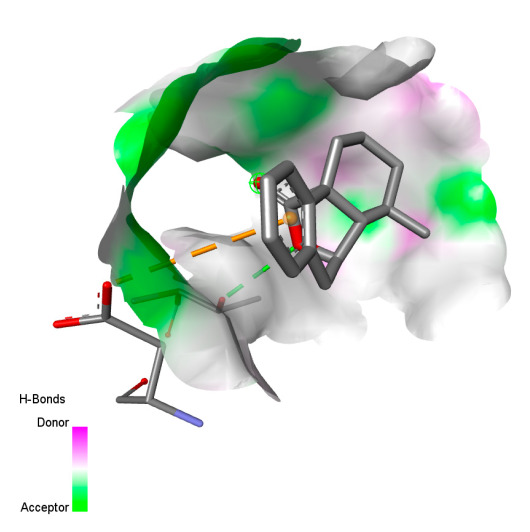	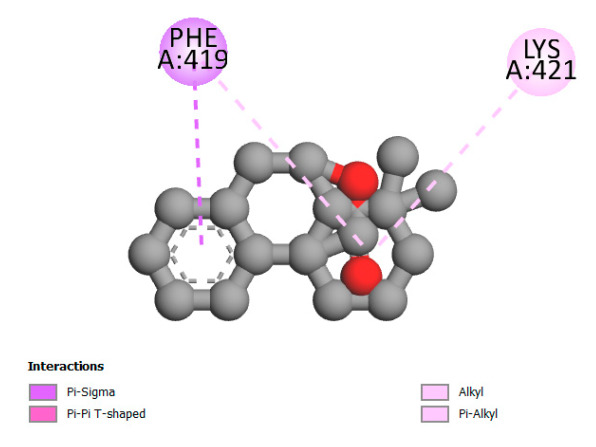	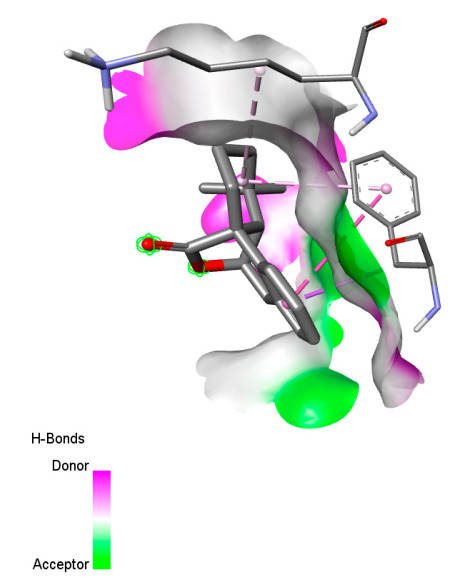
Salvianolic acid K	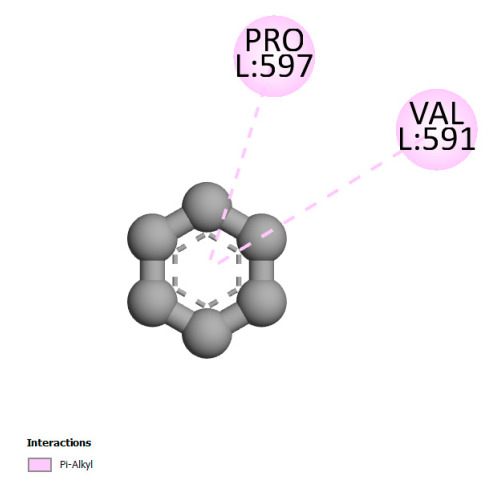	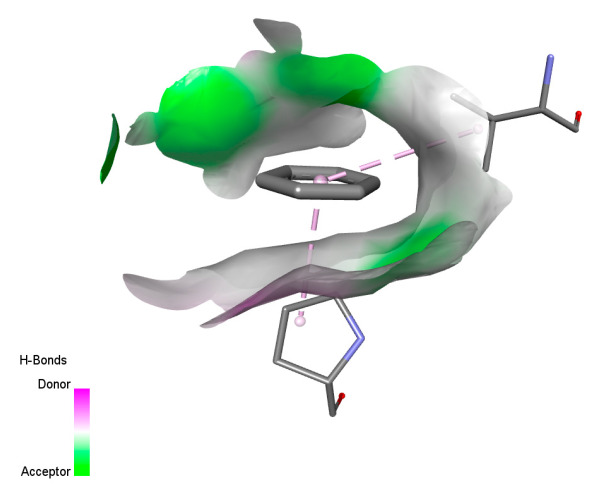	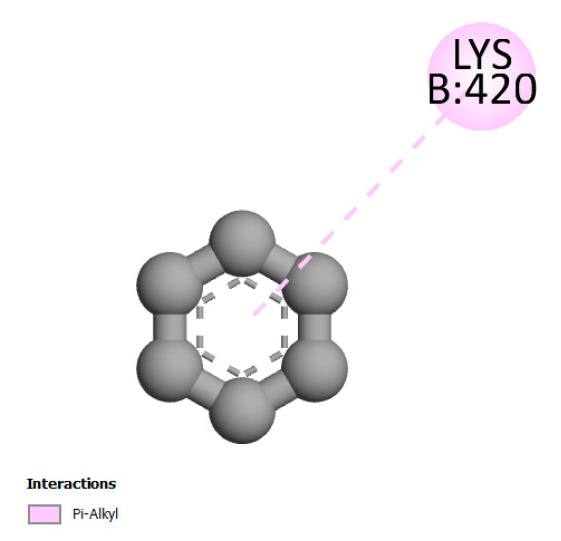	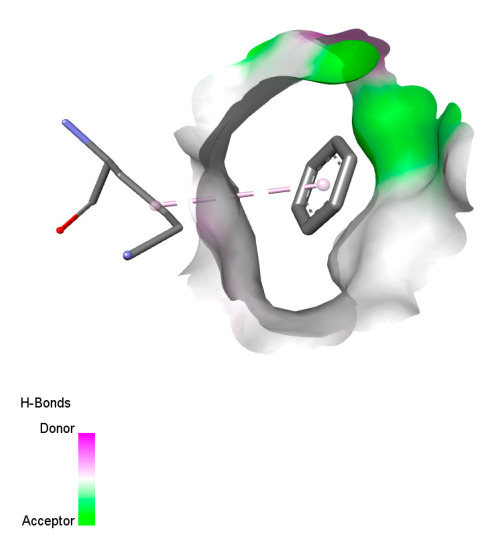

**Table 11:** Characteristics of the studied plant.

Plant Species	Vernacular	Harvest Site		Parts Used	Latitude (x)	Longitude (y)	Altitude (m)	Harvest Year
		Region	Locality					
*F. vulgare* Mill	Fenouil	Meknès	Ain jerry	Seeds	5°49′55″ O	33°85′86″ N	546	2024

**Table 12:** Key determination of the species.

Reign	Plantae
Kingdom	Fenouil
Class	Equisetopsida
Order	Apiales
Family	Apiaceae
Genus	*Foeniculum*
Species	*Foeniculum vulgare*

**Table 13:** Coding of extracts.

Extraction Method	Solvants	Codification
Decoction	Water	E (2)
Soxhlet	Ethanol/Water (70/30)	E (1)
	Water	E (0)

**Table 14:** List of bacterial strains tested with their references.

Bacterial Strains	References	Fungal Strains	References
*Enterobacter cloacae*	02EV317	*Candida albicans*	Ca
*Klebsiella pneumoniae*	3DT1823	*Candida dubliniensis*	Cd
*Escherichia coli* sauvage	3DT1938	*Candida tropicalis*	Ct
*Staphylococcus aureus* BLACT	4IH2510	*Candida parapsilosis*	Cpa
*Staphylococcus epidermidis*	5994	*Aspergillus niger*	AspN

**Table 15:** Protein targets and molecular docking parameters.

Activities	Targets	PDB ID	Grid Box Center Coordinates	Grid Box Size
Antibacterial activity	Beta-lactamase	7TI1	center_x = −56.105 center_y = 21.017 center_z = 50.112	size_x = 30 size_y = 22 size_z = 24
	DNA topoisomerase 4 subunit A	3RAE	center_x = −54.023 center_y = 68.181 center_z = −18.042	size_x = 22 size_y = 36 size_z = 34
	DNA gyrase subunit B	4DUH	center_x = 21.304 center_y = 12.134 center_z = 25.205	size_x = 24 size_y = 24 size_z = 22
	DIHYDROFOLATE REDUCTASE TYPE 1 FROM TN4003	2W9S	center_x = 6.027 center_y = −1.060 center_z = 30.037	size_x = 24 size_y = 28 size_z = 30
	tyrosyl-tRNA synthetase	1JIJ	center_x = −9.074 center_y = 18.180 center_z = 93.030	size_x = 24 size_y = 28 size_z = 30
	Transcriptional regulator TcaR	3KP5	center_x = −27.301 center_y = −30.531 center_z = −1.040	size_x = 26 size_y = 28 size_z = 24
	Ubiquinol--cytochrome-c reductase subunit	7RJB	center_x = 148.091 center_y = 127.219 center_z = 147.014	size_x = 24 size_y = 24 size_z = 28
	Lanosterol 14-alpha demethylase	5V5Z	center_x = −44.181 center_y = −14.027 center_z = 22.108	size_x = 28 size_y = 30 size_z = 36
	Candidapepsin-2	4YBF	center_x = 9.025 center_y = 1.007 center_z = 15.741	size_x = 26 size_y = 32 size_z = 34
	Structure of A. niger Fdc1	4ZA5	center_x = 19.027 center_y = 1.012 center_z = 19.118	size_x = 24 size_y = 24 size_z = 22
Antioxidant activity	Myeloperoxidase	5qj2	center_x = −49.012 center_y = 9.017 center_z = 29.115	size_x = 22 size_y = 28 size_z = 32
	Xanthine dehydrogenase/oxidase	3NRZ	center_x = 58.097 center_y = 3.009 center_z = 35.108	size_x = 22 size_y = 28 size_z = 32
	Cytochrome P450 2C9	1OG5	center_x = −38.207 center_y = 61.001 center_z = 27.024	size_x = 24 size_y = 22 size_z = 28
	Lipoxygenase-3	1N8Q	center_x = 26.027 center_y = 0.050 center_z = 16.130	size_x = 20 size_y = 28 size_z = 34
	NADPH oxidase	2CDU	center_x = 11.204 center_y = 1.035 center_z = 24.135	size_x = 20 size_y = 28 size_z = 34

## Data Availability

Data are contained within this article.

## Supplementary Materials

The following supporting information can be downloaded at: https://www.mdpi.com/article/10.3390/ijms26104499/s1.
