# Supplementary material for: Unveiling the Chemical Composition, Antioxidant, and Antimicrobial Potentials of Foeniculum vulgare Mill: A Combined In Vitro and In Silico Approach
Source: Int J Mol Sci. 2025 May 8;26(10):4499. doi: 10.3390/ijms26104499 (PMC12111592; doi:10.3390/ijms26104499)
Supplement: Supplementary file 1 [file ijms-26-04499-s001.zip › ijms-3596846-supplementary.pdf]

Table S1. Chemical composition of *F. vulgare* extracts by HPLC/UV-DAD.

N°	RT (min)	Compounds Identified	Structures	Classes	Exact Weights	[M-H]– (m/z)	Fragment Ions (m/z)	R.A (%)		
								E (0)	E (1)	E (2)
1	4,03	Caffeic acid 3-glucoside	$C_{15}H_{18}O_9$	Phenolic compound	342	341	341-179-149-131	0	1,33	1,82
2	4,31	Medioresinol	$C_{21}H_{24}O_7$	Lignan	388	387	207-179	0	1,21	7,35
3	4,74	Resveratrol	$C_{14}H_{12}O_3$	Polyphenol	228	227	227-185-143	0	0,45	3,19
4	5,21	Quinic acid	$C_7H_{12}O_6$	Phenolic acid	192	191	191 - 127	3,35	2,81	0
5	7,63	Embelic acid	$C_{17}H_{26}O$	Phenolic acid	294	293	293-184	0	0,69	6,32
6	8,49	Dihydrocaffeic acid	$C_9H_{10}O_4$	Phenolic acid	182	181	181-137	2,08	0	11,25
7	8,78	Emmotin H	$C_{15}H_{16}O_3$	Terpenoid	244	243	243-228-200-184	0	0	4,36
8	8,84	Syringic acid	$C_9H_{10}O_5$	Phenolic acid	198	197	179-135-123	1,06	3,05	0,66
9	9,03	Ascorbyl myristate	$C_{20}H_{34}O_7$	Carboxylic acid ester	386	385	385-323-291-171	1,04	0	0
10	9,16	Carnosine	$C_9H_{14}N_4O_3$	Dipeptide	226	225	225-179	0	0	5,42
11	9,98	3-O-Caffeoylshikimic acid	$C_{16}H_{16}O_8$	Phenolic acid	336	335	335-291-197	1,06	0,51	11,08
12	10,43	Rosmanol	$C_{20}H_{26}O_5$	Phenolic diterpene	346	345	345-283	0	1,29	8,08
13	10,85	Kaempferide	$C_{16}H_{12}O_6$	Flavonoid	300	299	217-149-107	0	0	2,82
14	11,11	Ferulic acid	$C_{10}H_{10}O_4$	Phenolic acid	194	193	193-161-133	0	0	1,2
15	11,28	Folic Acid	$C_{19}H_{19}N_7O_6$	Vitamin	442	441	293-119	1,82	0,56	1,84
16	12,33	Apigenin 7-methyl ether 5-(6''-malonylglucoside)	$C_{25}H_{24}O_{13}$	Flavonoid	532	531	531-369-269	2,8	0	0
17	12,51	Kaempferol 3-(6''-galloylgalactoside)	$C_{28}H_{24}O_{15}$	Flavonoid	600	599	599-447-419	0	0,57	0
18	12,79	Ferulic acid 4-glucuronide	$C_{16}H_{18}O_{10}$	Phenolic acid	370	369	369-193	3,97	0	0
19	13,15	6-Methylapigenin	$C_{16}H_{12}O_5$	Flavonoid	284	283	283-268-240	0	3,63	0
20	13,47	3-Feruloylquinic acid	$C_{17}H_{20}O_9$	Phenolic acid	368	367	367-175-115	0	0	1,14
21	14,24	(+)-Catechin 3-O-glucose	$C_{21}H_{24}O_{11}$	Flavonoid	452	451	451-271-255	0	0	1,26
22	14,27	δ-Tocopherol	$C_{27}H_{46}O_2$	Phenolic acid	402	401	401-175-135	0	1,22	0
23	14,55	Feruloyltartaric acid	$C_{14}H_{14}O_9$	Phenolic compound	326	325	193-149-134	2,66	0	0,54
24	14,74	Benzoic acid, 3,4,5–trihydroxy-2-oxo-1,3-propanediyl ester	$C_{17}H_{14}O_{11}$	Ester	394	393	393-319	0	0,55	0
25	14,97	Oleanic acid	$C_{30}H_{48}O_3$	Triterpenoid	456	455	455	0	0	0,43
26	15,43	(-) Epicatechin	$C_{15}H14O_6$	Flavonoid	290	289	245-205-179-125	0	0	2,91
27	15,56	Dihydro resveratrol	$C_{14}H_{14}O_3$	Phenolic compound	230	229	229-123	3,16	0	0
28	15,56	Salvianolic acid C	$C_{26}H_{20}O_{10}$	Phenolic acid	492	491	491-293	0	1,66	0

29	15,91	Naringenin	$C_{15}H_{12}O_5$	Flavonoid	272	271	271-245	0	0	1,96
30	16,29	Oleuropein aglycone	$C_{19}H_{22}O_8$	Terpenoid	378	379	197-158	4,42	1,16	1,67
31	16,21	Epigallocatechin gallate	$C_{22}H_{18}O_{11}$	Flavonoid	458	457	457-125	0	1,01	0
32	16,46	Dihydro Ferulic Acid 4-O-beta-D-Glucuronide	$C_{16}H_{20}O_{10}$	Phenolic acid	372	371	371-195-177	0	1,93	0
33	16,49	Rosmarinic acid	$C_{18}H_{16}O_8$	Phenolic acid	360	359	359-161-133	3,66	0	0
34	17,24	(+)-Catechin Hydrate	$C_{15}H_{16}O_7$	Flavonoid	308	307	307-289-179-125	1,61	1,08	0
35	17,35	Methyl rosmarinate	$C_{19}H_{18}O_8$	Polyphenol	374	373	373-179-135	2,14	0	2,14
36	17,63	Quercetin 3-O-xyloside	$C_{20}H_{18}O_{11}$	Flavonoid	434	433	433-301-271	0	0	1,58
37	17,96	Pinoresinol	$C_{20}H_{22}O_6$	Lignan	358	357	357-343-289	0	0	0,47
38	18,12	Chlorogenic acid	$C_{16}H_{18}O_9$	Phenolic acids	354	353	191-179	7,06	14,79	0,67
39	18,22	Resveratrol 3-glucoside	$C_{20}H_{22}O_8$	Polyphenol	390	389	389-227-185	5,42	2,28	0
40	19,24	Isochlorogenic acid A	$C_{25}H_{24}O_{12}$	Phenolic acids	516	515	515-353-179	7,53	5,98	0
41	19,24	Apigenin-7-O-glucoside	$C_{21}H_{20}O_{10}$	Flavonoid	432	431	431-385-207	0	0	0,84
42	19,58	Caffeic acid	$C_9H_8O_4$	Phenolic acid	180	179	179-135	1,91	1,05	0
43	19,96	Rhamnazin	$C_{17}H_{14}O_7$	Flavonoid	330	329	329-151	0	0,73	0,6
44	20,09	Catechin-4-ol 3-O-beta-D-glycopyranoside	$C_{21}H_{24}O_{12}$	Flavonoid	468	467	467-305-289-179	1,73	1,2	0
45	20,34	Ethyl p-methoxycinnamate	$C_{12}H_{14}O_3$	Carboxylic ester	206	205	205-161	0	0	0,66
46	20,61	Azelaic acid	$C_9H_{16}O_4$	Fatty acid	188	187	169-125	0	0	1,04
47	21,04	Linoleic Acid	$C_{18}H_{32}O_2$	Fatty acid	280	279	279-127	0	0	0,63
48	22,4	Rutin	$C_{27}H_{30}O_{16}$	Flavonoid	610	609	609-301	3,10	3,16	0
49	22,5	Shogaol	$C_{17}H_{24}O_3$	Phenolic compound	276	275	275-189-135	1,3	2,21	0
50	26,36	Quercetin-3-D-xyloside	$C_{20}H_{18}O_{11}$	Flavonoid	434	433	433-301-271	3,18	6,73	0
51	23,61	Quercetin-3-glucuronide	$C_{21}H_{18}O_{13}$	Flavonoid	478	477	477-301-151	2,7	13	0
52	25,81	Butyl ferulate	$C_{14}H_{18}O_4$	Phenolic acid ester	250	249	249-193-134	14,33	0	0,42
53	23,22	Catechin 3-rhamnoside	$C_{21}H_{24}O_{10}$	Flavonoid	436	435	435-289-179	0	2,16	0
54	23,49	Alizarin	$C_{14}H_8O_4$	Quinone	240	239	239-211-167	0	0	0,49
55	23,72	Apigenin	$C_{15}H_{10}O_5$	Flavonoid	270	269	269-227-159	6,63	0	0
56	25,29	Ethyl chlorogenate	$C_{18}H_{22}O_9$	Polyphenol	382	381	381-191-179	0	0	2,51
57	25,4	Pinobanksin 3-O-propanoate	$C_{18}H_{16}O_6$	Flavonoid	328	327	327-255	0	0	4,46
58	25,45	Emmotin A	$C_{16}H_{22}O_4$	Sesquiterpenoid	278	277	277-243	0	5,42	0
59	25,51	Caffeoyl-feruloyltartaric acid	$C_{23}H_{20}O_{12}$	Phenolic acid	488	487	487-443-293	4.0	0	0,53
60	25,61	Rhamnetin 3-rhamnoside	$C_{22}H_{22}O_{11}$	Flavonoid	462	461	461-315-271	0	2,39	0

61	25,77	Salvianolic acid K	$C_{27}H_{24}O_{13}$	Polyphenol	556	555	555-493-313-179	0	6,71	0
62	25,81	Catechin 7-O-apiofuranoside	$C_{20}H_{22}O_{10}$	Flavonoid	422	421	421-289-137	0	0	2,81
63	25,98	7-Methoxy-2-methylisoflavone	$C_{17}H_{14}O_{3}$	Flavonoid	266	265	265-237-179	2,65	7,25	0
64	26,18	Kaempferol	$C_{15}H_{10}O_{6}$	Flavonoid	286	285	285-151	0	0	0,48
65	27,84	Butyl chlorogenate	$C_{20}H_{26}O_{9}$	Polyphenol	410	409	409-191-135	3,64	0,5	0
